# On the Emergence of Phonological Knowledge and on Motor Planning and Motor Programming in a Developmental Model of Speech Production

**DOI:** 10.3389/fnhum.2022.844529

**Published:** 2022-05-12

**Authors:** Bernd J. Kröger, Trevor Bekolay, Mengxue Cao

**Affiliations:** ^1^Department of Phoniatrics, Pedaudiology, and Communication Disorders, Medical Faculty, RWTH Aachen University, Aachen, Germany; ^2^Applied Brain Research, Waterloo, ON, Canada; ^3^School of Chinese Language and Literature, Beijing Normal University, Beijing, China

**Keywords:** motor planning, motor programming, speech production, developmental model, phonological knowledge, sensorimotor system, cognitive-linguistic system

## Abstract

A broad sketch for a model of speech production is outlined which describes developmental aspects of its cognitive-linguistic and sensorimotor components. A description of the emergence of phonological knowledge is a central point in our model sketch. It will be shown that the phonological form level emerges during speech acquisition and becomes an important representation at the interface between cognitive-linguistic and sensorimotor processes. Motor planning as well as motor programming are defined as separate processes in our model sketch and it will be shown that both processes revert to the phonological information. Two computational simulation experiments based on quantitative implementations (simulation models) are undertaken to show proof of principle of key ideas of the model sketch: (i) the emergence of phonological information over developmental stages, (ii) the adaptation process for generating new motor programs, and (iii) the importance of various forms of phonological representation in that process. Based on the ideas developed within our sketch of a production model and its quantitative spell-out within the simulation models, motor planning can be defined here as the process of identifying a succession of executable chunks from a currently activated phoneme sequence and of coding them as raw gesture scores. Motor programming can be defined as the process of building up the complete set of motor commands by specifying all gestures in detail (fully specified gesture score including temporal relations). This full specification of gesture scores is achieved in our model by adapting motor information from phonologically similar syllables (adapting approach) or by assembling motor programs from sub-syllabic units (assembling approach).

## Theoretical Background

### The Models of Speech Production and Speech Perception Influencing Our Model Sketch

The process of speech production can be subdivided in concept preparation, lexical selection, morphological and phonological encoding, phonetic encoding, and articulation (Levelt et al., 1999). In a word production task, concept preparation is the activation of a lexical concept, followed by selecting its lemma and subsequently by retrieving its phonological form. It is emphasized by Levelt et al. (1999) that morphemes and not syllables are stored in the mental lexicon. Thus, lexical processing is followed by syllabification. Subsequently, syllables are encoded phonetically by specifying a gestural score (ibid., see Browman and Goldstein, 1992 for defining gestural scores for lexical units like monosyllabic words) and thus by specifying basic control units for the articulatory execution of the syllable under production. In parallel to the mental lexicon as the central higher-level knowledge repository, Levelt and Wheeldon (1994) and Levelt et al. (1999) postulate a mental syllabary as a storage for highly overlearned gestural patterns. These “ready-made” gestural scores or patterns are assumed to be stored within the mental syllabary of a speaker for all frequently used syllables of the speaker, and it is assumed that these patterns can be directly accessed and executed by the articulatory system.

The mental syllabary as introduced by Levelt and Wheeldon (1994), Levelt et al. (1999), and Cholin et al. (2006) is a repository for motor programs. While the motor programs of low-frequency syllables of a language are assumed to be calculated or constructed “on-line,” the mental syllabary is hypothesized to provide motor programs as “pre-compiled gestural scores” for high-frequency syllables. Moreover, it is assumed that the storage of motor programs does not overload the mental or neural capacity of the brain because only about 500 syllables can be labeled as high-frequency syllables for example in English, Dutch, or German. In these languages, 500 syllables make up only 5% of the entire syllable inventory, but these 500 syllables are sufficient for producing about 80% of all utterances in these languages (Schiller et al., 1996).

Beside storing execution-related neural representations like “motor programs” it can be assumed that auditory as well as somatosensory forms are stored in the mental syllabary as well (Kröger et al., 2019). This assumption is in accordance with the DIVA model of speech production introduced by Guenther (2006) and Guenther et al. (2006). Here, motor representations (motor commands) are stored for speech items in parallel to their sensory target representations (auditory and somatosensory states) in order to allow a sensory driven control (feedback control) during feedforward execution of a speech item. Thus, Guenther’s DIVA model (Directions Into Velocities of Articulators; Guenther, 2006; Guenther et al., 2006; Guenther and Vladusich, 2012; Kearney and Guenther, 2019) differentiates a feedforward and a feedback control subsystem. Production starts with the activation of a speech item in the “speech sound map,” which subsequently activates a set of motor commands passing the feedforward control system, which then activates a target in the motor map, here called “articulatory velocity and position map.” Activation patterns in this map directly result in articulator movements. In parallel the activation of a speech item in the speech sound map leads to a co-activation of an auditory and somatosensory target state for that speech item. During the production process, the activated sensory target states are compared with its sensory feedback states. In case of divergence, feedback commands (i) for on-line correcting the current production or (ii) for a later offline correction are generated and forwarded to the motor map for modifying execution. Thus, motor commands and the associated sensory target states can be updated with each production trial if necessary. Bohland et al. (2010, p. 1508) interpret the speech sound map as compatible with Levelt’s mental syllabary. It should be noted that the DIVA model undergoes (i) a babbling training process which provides continuous mappings between sensory and motor states and later (ii) an imitation training process in order to acquire motor representations for specific speech items like words or short phrases which are stored in the speech sound map. Imitation learning depends on knowledge concerning sensory-to-motor relations in order to generate first motor representations (first motor commands) for the speech item under imitation as well as for calculating the direction of further alterations of the motor representation of a speech item in order to approximate its acoustic target.

In parallel to the syllabification process as described by Levelt et al. (1999), Bohland et al. (2010), Guenther (2016), and Miller and Guenther (2021) propose a process for the division of the phonological sound sequence in executable speech items (chunks), for which sensorimotor programs already exist. This process is implemented in the GODIVA-model (Gradient Order DIVA model, Bohland et al., 2010) which differentiates a planning loop and a motor loop. The planning loop comprises a phonological content buffer and a sequential structure or structure frame buffer. The motor loop comprises the (speech) initiation map and the speech sound map. While the motor loop directly initiates the chain of sensorimotor programs (executable gesture scores) at the level of the speech sound map, the planning loop parses the incoming phonological sound sequence with respect to these executable chunks and selects chunks for later initiation by the motor loop. By activating potential syllabic chunks, which fit parts of the current sound chain, chunks of phonological sound sequences are selected and executed. Bohland et al. (2010) describe this process as an interaction or interfacing of selected phonological codes with “an elaborated speech sound map” to select best matching sensorimotor programs for execution (ibid., 1509). Here the speech sound map is interpreted as a neural buffer from which sensorimotor programs for high-frequency syllables can be initiated directly in full, whereas the sensorimotor programs of infrequent syllables must be assembled from smaller, e.g., phoneme-sized units (ibid., p. 1509 and see dual route approach, Varley and Whiteside, 2001) before they can be initiated and executed. The assembly process is later concretized by Bohland et al. (2010) by stating, that a phonological word to be produced can be effectively “spelled out” during production using motor programs for the individual phonemes (ibid., p. 1512). Thus, motor plans are available for whole syllables on the one hand but on the other hand motor plans of (new) syllables can be generated “using a sequence of smaller stored programs corresponding to the syllables’ individual phonemes” (ibid 1521). Thus, GODIVA stores motor plans of frequent syllables as well as motor plans for sub-syllabic phoneme-sized units within the speech sound map.

The DIVA model already stresses the importance of somatosensory and auditory feedback in speech production. While somatosensory feedback always stems from self-perception, auditory perception is self-perception as well as perception of other’s speech (auditory input from communication partners). The process of auditory speech perception can be subdivided in two routes, an auditory-conceptual (ventral) and an auditory-motor (dorsal) route (Hickok and Poeppel, 2007, 2016). The dorsal route activates appropriate motor representations and somatosensory representations if an auditory speech signal is processed (cf. sensorimotor integration; Hickok et al., 2011). The functional processing steps in the speech perception and speech processing model introduced by Hickok and Poeppel (2007, 2016) are spectro-temporal acoustic signal analysis followed by phonological processing. Subsequently the perceptual pathway separates in the dorsal stream which activates the motor network via a sensorimotor interface and in the ventral stream activating the lexical and combinatorial (conceptual) network.

One of the goals of this paper is to differentiate motor planning and motor programming as well as to define functional aspects of motor planning and motor programming. Our approach is based on already published concepts. (i) In the GODIVA model a phonological chain processing or selection process is separated from motor program initiation and execution (Bohland et al., 2010, p. 1512). (ii) Riecker et al. (2005) separate a cerebral motor preparation and a motor execution loop for speech production based on fMRI experiments. Because the task here was a simple syllable repetition task, preparation here comprises activation of motor programs but not motor planning processes. (iii) A four-level model focusing on the differentiation of planning and programming is introduced by van der Merwe (2021). Here a differentiation of linguistic symbolic planning, motor planning, motor programming and execution is postulated. While linguistic planning activates a phonemic representation (lexical and grammatical processing and syllabification), the motor planning module takes phonological code as input and “assigned properties amenable to a motor code” (ibid., p. 404). A set of motor commands is activated as output of the motor planning module, mainly specifying phonological-phonetic segmental features (ibid., p. 409). The motor programming module now uses motor plan information as input and outputs fully specified spatiotemporal articulatory movement information in form of muscle-specific motor programs. Motor programs here can be defined for whole syllables but as well for sub-syllabic units like segments or gestures.

### Early Phases of Speech Acquisition and Models of Speech Learning

The newborn starts to produce speech-like vocalic sounds, also called proto-vowels, at the age of about 3 months. It produces first canonical babbling patterns, also called proto-syllables or proto-CV patterns comprising proto-consonants (proto-C) and proto-vowels (proto-V), at the age of about 7 months. Language specific syllable productions start at about 10 months and first words are produced at about 12 months (Kuhl, 2004). The well-known fact that perception precedes production is underpinned by the fact that speech-specific phonetic contrasts can already be discriminated directly after birth and language specific perception of vowels already starts with 6 months. Recognition of language specific sound combination starts with 9 months (ibid.). By 18 months of age, 75% of typically developing children understand about 150 words and can successfully produce 50 words in case of American English (Kuhl, 2004, p. 834, citing Fenson et al., 1993). Moreover, the role of social interaction as occurring for example in the case of joint attention to an object is an important vehicle for word learning (e.g., Lytle and Kuhl, 2017).

Thus, the transition from newborn’s first vocalizations like crying, like production of vegetative sounds, and like first non-cry phonations toward the production of speech-like vowels including speech-like phonation (i.e., proto-vowels) and the transition from gooing and marginal babbling, both consisting of primitive tongue and lip movements toward canonical babbling occurs within the first 6–9 months of lifetime (Oller, 2000; Buder et al., 2013). Canonical babbling comprises the production of proto-syllables consisting of already well-formed consonantal closures and vocalic openings accompanied by speech-like phonation. It has been shown by means of computer simulations how canonical babbling emerges from earlier babbling stages and from pre-speech vocalizations by using reinforcement learning (reward-modulated learning, see Warlaumont and Finnegan, 2016). Here a reward is given if a new vocalization produced by the infant (by the model) is acoustically more salient than vocalizations produced earlier and productions which are accompanied by a caretaker’s reward are stored and reproduced more frequently. These simulations indicated that pure vocalic sounds are auditorily less salient than speech sounds which include vocal tract closures and releases of these closures, here labeled as “syllabic sounds.” The simulation experiments indicate that the frequency of canonical babbling (i.e., the frequency of auditory salient events) increases during ongoing reinforcement learning.

A further model of speech learning comprising the babbling and imitation phase is introduced by Kröger et al. (2009), Kröger and Cao (2015), and Kröger et al. (2019). Here, two self-organizing neural maps, i.e., a phonetic and a semantic map form the center of the speech processing neural network. The semantic map realizes the center of the cognitive-linguistic model part and the phonetic map realizes the center of the sensorimotor or phonetic model part. Babbling starts with a set of proto-syllables (pre-linguistic items) and proceeds toward learning of language specific sets of V-, CV-, VC-, and CCV-syllables. This babbling training leads to the development of the phonetic self-organizing map (SOM) which contains basic auditory-to-motor knowledge in order to enable imitation (Kröger et al., 2009). Imitation training leads to an advancement of this map. After imitation training the phonetic map is able to activate motor and sensory states for all syllables, trained so far. In parallel, imitation training leads to a buildup of the semantic SOM in the cognitive-linguistic part of the model (Cao et al., 2014; Kröger and Cao, 2015). Simulation experiments were carried out for learning or training a model-language comprising of about 70 monosyllabic words. After learning, word production can be simulated by activating a word node (a model neuron) within the semantic map which co-activates sensorimotor nodes within the mental syllabary and thus co-activates motor and sensory states for each selected word.

In this approach, the main result of babbling training is the association of auditory, somatosensory, and motor states of proto-syllables within the self-organizing phonetic map. In addition, an ordering of proto-syllables appears with respect to phonetic features like vocalic high-low, front-back or consonantal manner and place of articulation. The main result of imitation training is that these proto-syllabic motor and sensory states represented in the phonetic map during babbling training now are more and more shaped with respect to specific syllable realizations of the target language. Moreover, imitation training leads to an association of words with those syllables which are already represented by the phonetic map. This allows the extraction of phonological features and of phonological knowledge from the ordering of syllables within the phonetic map because this ordering which has already been established during babbling will remain and will be expanded during imitation training (Kröger and Cao, 2015; Kröger et al., 2019).

A further simulation approach for speech learning using SOMs has been proposed by Li et al. (2004). In contrast to the models described above this approach does not include acoustic or motor information. Here, a segmental feature description of speech items is used as phonological input information and two different semantic feature descriptions are used as semantic input representations. This approach models the early lexical development up to a lexicon size of about 500 words. The model starts with imitation of speech items. In this approach, the learner (the model) already has available phonological knowledge including the phoneme repertoire of the target language. On this basis the model is capable to simulate learning effects occurring during lexical development like lexical confusion effects occurring in early vocabulary learning as well as age-of-acquisition effects.

### The Emergence of Phonological Representations

The models described so far differ in introducing a level of phonological representation. Because a phonological representation is language-specific this representation emerges during speech acquisition. During the imitation phase first phonetic features and broad categorizations like labial, apical vs. dorsal place of articulation, like voiced vs. voiceless and like nasal vs. oral sound production result from differentiating babbling items. Moreover, proto-vocalic productions with palatal, velar and pharyngeal narrow passages lead to phonetic vowel categories like [i], [a], and [u], and thus to phonetic features like high-low front-back. These broad categorizations and its resulting phonetic features can be interpreted as precursors of language-specific phoneme sets and phonological features. These initial processes are followed by a complex process of tuning the perceptual categories and the articulation of speech sounds in a language specific direction up to an age of 6 years (Gervain and Mehler, 2010; Redford, 2019). As an example, in case of English and Dutch, most language specific vowels are learned at about 3 years of age, and most consonants already at about 4 years of age, except some fricatives. Complex consonant clusters develop between 4 and 6 years of age (Priester et al., 2011). But typical patterns of articulatory alterations or simplifications like gliding, stopping, epenthesis, cluster simplification can still be observed until school-age years even in normally developing children (Redford, 2019, p. 2952; citing Stoel-Gammon and Dunn, 1985, pp. 43–46). Thus, it can be assumed that phonological knowledge like the notion of phonemes as well as of distinctive features emerges over the entire time span of speech acquisition (emergentist model, e.g., Menn and Vihman, 2011, continuity hypothesis, e.g., Fikkert, 2007).

### Segmental Versus Gestural Approaches

Beside developmental approaches supporting segmental concepts and introducing a phonological level of representations, Redford (2019) suggests a developmental approach based on holistic motoric representations or action schemas for the representation of words. Here, four major developmental milestones are postulated: (1) A perceptual-motor map for associating perceptual and motor forms of syllable-sized speech items already develops during the pre-speech period and continuously develops during speech learning. (2) During imitation, perceptual word forms (referential adult productions) are the starting point for word learning. Action schemas are now influenced and refined by language-specific imitation of syllables. At about 12 months of age a stable perceptual lexicon of about 100 words is established. Motor routines or action schemas now are associated with first words using the already existing perceptual-motor map. (3) Perceptually based control becomes more and more important at about 18 months of age. While productions are motorically constrained during the babbling phase, perception now forces articulation to widen and to refine the movement repertoire dramatically. (4) While the third phase marks the onset of perceptual control and while speech learning is mainly communication-driven in this third developmental stage the fourth stage emphasizes self-perception. Redford (2019) states that “speech production does not become adultlike until children begin to externally monitor their own speech and consciously recognize its divergence from (chosen) adult norms” (ibid. p. 2956). Thus, the reward in reinforcement learning during imitation now switches from external reward given by communication partners toward self-judgment of the phonetic quality of word production.

Moreover Redford (2019) separates information processing approaches and ecological dynamics approaches. In the first category phonological representations mediate between perception and production. Here the sequencing of discrete elements like phonemes plays a central role and discrete steps are needed to translate discrete symbolic representations into action plans (e.g., Levelt et al., 1999). The second category represents the non-segmental concepts like that of Articulatory Phonology (Browman and Goldstein, 1992; Goldstein et al., 2006). Here, the segmental or phonemic level is avoided by introducing gestures as an action unit on the one hand and as a distinctive phonological unit on the other hand. Moreover, this approach allows a direct linking of lexical forms to action forms (for a definition of “action units” see the task dynamics concept as introduced by Saltzman and Munhall, 1989). Gestures (or actions) are dynamically defined target-directed movement units, and the temporal coordination of gestures is quantified by using a concept of phasing which is based on intrinsic time scales (Goldstein et al., 2006). The minimal unit of speech production (molecule) described in the framework of Articulatory Phonology is the syllable or the one-syllabic word while gestures are seen here as minimal production units (atoms).

The model described in this paper assumes the neurobiological reality of gestures as well as of phonemes and distinctive features as units of speech processing (production and perception). While gestures appear to be the adequate units for describing speech during early phases of speech learning (during babbling and early phases of imitation) as well as later during adult speech production, it is assumed in our approach that an intuitive awareness of distinctive features, of phonemes and of syllable structures like CV, CVC, or CCV establishes during the time span of speech acquisition (Grunwell and Yavas, 1988; Levelt and van de Vijver, 2004). Thus, we use the concept of gestures, gesture scores and of intrinsic timing of gestures mainly as a concept for describing proto-syllables as well as language-specific syllables. But during imitation training gestures can be defined more and more by distinctive features. Thus, a glottal opening/closing gesture for example represents the feature unvoiced/voiced; a labial/apical/dorsal closing gesture represents the feature “place of articulation.” A closing/near-closing gesture represents different values for the feature “manner of articulation” etc. (Kröger and Birkholz, 2007). Beside this phonological aspect of gestures, the motor aspect of gestures and gesture scores can be implemented by introducing syllabic neural oscillators for defining the temporal coordination of gestures and by introducing gesture neural oscillators for defining the spatio-temporal aspects for the realization of each gesture within a gesture score (Kröger et al., 2021).

### Goals of This Paper

It is the goal of this paper to formulate a sketch for a model of speech production which comprises the cognitive-linguistic as well as the sensorimotor part of speech production, which includes developmental aspects of speech production, and which emphasizes the emergence of segmental or gestural phonological representations as an important part of developmental processes (i.e., of speech acquisition). Our model sketch can be interpreted as a theory of speech production and speech acquisition and parts of our model sketch are underpinned by quantitative computer simulations. (i) A conventional connectionist model (model 1, Kröger et al., 2019) is used for illustrating the buildup of the mental syllabary during early processes of speech acquisition, i.e., babbling and imitation. (ii) A spiking neuron approach including a detailed modeling of time-dependent neural processes (model 2, Kröger et al., 2020) is used to illustrate different processes of motor programming. Thus, two different computer-implemented models are used here in order to illustrate different aspects of speech acquisition and speech processing. While conventional connectionist approaches are able to highlight processes of increasing self-organization in neural networks, which are based on learning as they appear during speech acquisition (see e.g., the SOMs approaches of Li et al., 2004; Kröger et al., 2014), contemporary spiking neuron approaches are able to combine cognitive discrete neural processes (here mainly lexical processes) with sensorimotor processes and these models are able to model temporal aspects of neural and peripheral processing in a straight forward way (see e.g., the large scale neural model of Eliasmith et al., 2012).

## The Sketch for a Model of Speech Production

Our model of speech processing separates modules or sub-networks for processing (production or perception) and for the storage of knowledge and skills (neural repositories). Linguistic knowledge is stored in the *mental lexicon* (repository for words, lemmas, and phonological word forms) and in a grammatical rule component (not implemented thus far). Phonetic knowledge and sensorimotor skills are stored in the *mental syllabary* (repository of motor and sensory forms of already learned syllables).

While a level of phonological representations is of central importance in many production and perception models, this level emerges in our model during the entire process of speech acquisition. For production the *phonological form* represents the output level for the cognitive-linguistic part of the model (e.g., Levelt et al., 1999) and it represents the input level for the phonetic-sensorimotor part of the model (e.g., Guenther, 2006). For perception the phonological form represents an intermediate level arising between the module of spectro-temporal analysis and the module of lexical processing in the ventral stream of speech perception as well as between the module of spectro-temporal analysis and the sensorimotor interface in the dorsal stream of speech perception (e.g., Hickok and Poeppel, 2007, 2016). Three developmental phases can be separated in our modeling approach. (i) *babbling* for processing of pre-linguistic proto-speech items (starts at an age of 3 months) and for developing an early version of the mental syllabary, i.e., a phonetic map; (ii) *imitation* as an early stage of language-specific speech *processing* (starts at an age of 6 months and overlaps with babbling) for further developing the mental syllabary and for developing the mental lexicon as well as phonological knowledge; and (iii) *adult speech* processing as a processing stage occurring after speech acquisition (starts at about 6 years of age) using mental syllabary and mental lexicon.

### Babbling Stage of the Model Sketch

Babbling allows the model to learn auditory-to-motor relations from pre-linguistic proto-speech items and allows the model to build up a preliminary sensorimotor skill repository (called phonetic map) for storing the motor states, the somatosensory states, and the auditory states of already trained proto-speech items. The sensory and motor states are associated with each other for each trained proto-speech item. In our model sketch (as well as in Kröger et al., 2009) neural buffers are defined for hosting motor forms (*motor states*), and sensory forms (*auditory and somatosensory states*) of speech-like items. These buffers are connected to a neural SOM, called phonetic map, which is capable to activate each proto-speech item by activating its motor state and by co-activating its sensory states within the appropriate state buffers. Each proto-speech item is represented within the phonetic map by a specific neural activation pattern, which can be represented in a simple connectionist approach – in which the phonetic map is represented by a SOM – by the activation of a single node within the phonetic map (ibid.). Training is done here by babbling proto-V and proto-CV items over the whole range of vocalic vocal tract states and by combining these vocalic states with labial, apical, and dorsal closing gestures. An analysis of the resulting topology of the trained phonetic map reveals that these trained proto-speech items are ordered with respect to auditory as well as to somatosensory and motor features.

**FIGURE 1:**
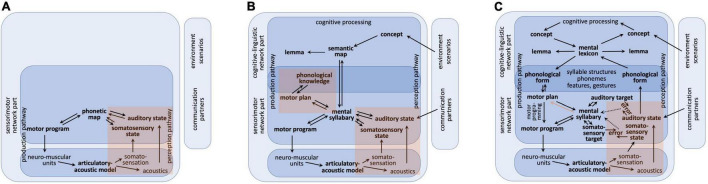
Sketch for a model of speech production within three different developmental phases. **(A)** Babbling, **(B)** imitation, **(C)** complete production-perception network; red regions: sensory feedback pathways leading to somatosensory and auditory states **(A–C)** and leading to phonological knowledge mainly during imitation phase **(B)**; red arrow [in part **(C)** only]: pathway for forwarding information concerning existing motor programs from mental syllabary toward the motor planning level mainly during adult speech production. This leads to a choice between direct route (activation of an existing motor program via mental syllabary) and programming route (full specification of gesture score at motor plan level followed by generating a motor program for a syllable; see text).

In our modeling approach, a babbling trial starts with the activation of a motor program for a proto-speech item (motor program in Figure 1A). The subsequent neuromuscular activation pattern leads to specific movements and displacements of speech articulators and this resulting articulatory pattern leads to an acoustic speech signal which is generated from the articulatory-acoustic vocal tract apparatus (Figure 1). The somatosensory (tactile and proprioceptive) feedback signals stemming from the articulatory movement pattern as well as the auditory feedback signal leads to neural activations in the appropriate sensory state buffers and to an activation at the level of the phonetic map (Figure 1). This temporally overlapping activation of a motor state and its resulting feedback sensory states for each trained proto-speech item leads to an association of sensory and motor states at the level of the phonetic map. If a proto-speech item has been produced several times (about 10 times per item, see Kröger et al., 2009, p. 802: 5000 training steps for 465 CV-training items and 5000 training steps for 500 V-training items) its motor and sensory states are associated and this item is stored or represented within the phonetic map.

Babbling ends with *a set of learned sensory-motor relations* (sensory comprises auditory and somatosensory) by storing auditory, somatosensory, and motor patterns for a variety of babbled proto-speech items. These auditory-to-motor relations are needed for later imitation training.

The somatosensory representation can be interpreted in our model as a simplified representation of motor states. While a motor program includes a detailed pattern of neural activations over time for all neuromuscular units of all articulators, the somatosensory state directly refers to articulation and thus allows a more direct and probably simplified description of an articulatory pattern.

The auditory state map is quantified in our approach by specifying the formant patterns of a syllable, which are the F1-, F2-, and F3-trajectories within the frequency-time space (Kröger et al., 2009; Cao et al., 2014; Kröger et al., 2014). The motor state map is quantified by listing the activation patterns of all gestures representing a syllable (Kröger et al., 2021). The somatosensory state map is quantified by specifying the movement patterns for the degree of lips opening, tongue tip, tongue body, and lower jaw elevation (Kröger et al., 2019).

An advantage of using articulatory gestures as basic production units is that proto-syllables can be interpreted as being composed of discrete units (i.e., raw gestures). These raw gestures already exist at very early stages of speech acquisition (i.e., the beginning of babbling) and the set of raw gestures can be used to define a set of distinctive features: (i) a proto-syllable contains at least a vocal tract opening gesture and/or contains a closing gesture (feature proto-V vs. proto-C). This allows a separation of proto-V and proto-CV syllables; (ii) the articulator of a closing action separates labial, apical, or dorsal proto-consonants (feature: place of articulation = labial/apical/dorsal); (iii) the absence vs. presence of a glottal opening gesture separates voiced vs. voiceless proto-consonants as part of the proto-syllable (it can be assumed that this feature voiced/voiceless develops later during babbling and is refined during imitation phase); (iv) the absence vs. presence of a velopharyngeal opening gesture separates nasal vs. oral consonants (it can be assumed that the feature nasality as well develops later during babbling and imitation phase). It should be noted that the timing of all gestures as well as their targets are still raw (i.e., proto-gestures) and not fine-tuned with respect to any target language at this stage of speech learning.

### Imitation Stage of the Model Sketch

The model is now capable for imitation of language-specific speech items picked up from external speakers (caretakers or communication partners, see Figure 1B) because a preliminary knowledge base for auditory-to-motor state mappings has been established during babbling as part of the phonetic map. An incoming auditory pattern, for example a word, which is tried to be imitated by the child, activates an auditorily similar babbling item available in the phonetic map. Because the activated babbling pattern only approximates the incoming auditory patterns the motor program of a babbling pattern is systematically varied during imitation until a word production is rewarded (i.e., understood) by the communication partner. This allows the model to adapt link weights between phonetic map and state maps in order to be able to reproduce this new or refined motor state and its appertaining feedback sensory states in the phonetic map as a preliminary word realization.

Here we assume that imitation of a word – which activates a node in the self-organizing phonetic map – always co-activates a node in the self-organizing semantic map and thus leads to an activation of the word within the semantic map as well as to an activation of its phonetic realization within the phonetic map. Therefore, we presume *communication scenarios* in which the child points or focuses on an object like a ball, then looks at the caretaker and thus forces the caretaker to produce that word. Thus, during the period of actively imitating a specific word, the cognitive-linguistic as well as the sensorimotor part of the model is involved which leads to a bilateral activation and association of a specific neural state within the self-organizing semantic and within the self-organizing phonetic map (Figure 1B; and see Kröger et al., 2011).

Imitation of a word may occur many times during the imitation phase which leads to an increase in approximating the correct phonetic realization of the word. This process is called *refining, tuning, and differentiating of motor patterns* (cf. Nittrouer, 1995). In our modeling approach this process expands the set of already stored pre-linguistic sensorimotor items toward *a set of language-specific syllable realizations*. The phonetic map can now be relabeled as mental syllabary (Figure 1B). The nodes of the mental syllabary represent language-specific frequent syllables (Kröger et al., 2009; Kröger and Cao, 2015; Kröger et al., 2019).

As a result of learning during the babbling phase basic proto-vocalic and proto-consonantal gestures appear within raw motor programs (i.e., within raw gesture scores). Later during imitation training gesture scores and the appropriate motor programs can be differentiated not only with respect to basic types of gestures like closing and opening gestures or with respect to different gesture-executing articulators like lips, tongue tip and tongue dorsum but in addition with respect to *segmental features* like voicing and nasality because now the language-specific temporal location of proto-vocalic, proto-consonantal, velopharyngeal and glottal opening and closing gestures is learned. In our model sketch this type of motor representation is called *motor plan* or *raw gesture score.* Motor plans are available at the end of the babbling phase and thus during the entire imitation phase (motor plan level, Figure 1B). The process of refining, tuning and differentiation of motor plans and motor programs during the imitation phase leads to a set of language-specific gestures and features. This can be interpreted as emergence of phonological knowledge.

Thus, learned items (motor plans and motor programs and their sensory correlates) at the end of imitation can already be ordered with respect to phonological categories of the target language and thus can be interpreted as realizations of (language-specific) syllables (Kröger et al., 2019). Realizations of syllables belonging to the same phonemic state appear to build “phoneme regions” within the SOM (ibid.). Specific regions appearing within the SOM of the mental syllabary can now be labeled as phonological distinctive regions, because the syllable realizations stored here are linked with words and thus with meanings. The model develops *phonological knowledge* concerning (i) syllable structures, (ii) sound types (e.g., vowels vs. consonants) and (iii) sound features (e.g., place and manner of articulation). The syllable can now be specified by a bundle of features for the articulatory closing and opening portions occurring within the syllable and thus different types or categories of consonants and vowels can be distinguished and it can be assumed that the speaker (the model) now is aware of a sequence of different segmental categories (which can be labeled as a sequence of phonemes at the motor plan level). The corresponding motor plan state is labeled as “raw gesture score”.

The step from imitation phase (Figure 1B) toward the adult production-perception model (Figure 1C) is done now by including a level of phonological representations (based on the phonological knowledge acquired during imitation) as a concrete neural state level within our model. It can be assumed that the neural structure for this neural state level is already defined within the developing neural network laid out for (later) speech processing and this structure starts growing during the imitation phase of speech acquisition (Zhang and Wang, 2007).

This phonological level is part of the top–down processing of speech production (from lexical output toward motor plan specification) and of the bottom-up-processing in speech perception (from auditory form to lexical processing) in the adult speech processing model. Moreover, the adult production-perception model includes additional processing steps at the cognitive level based on knowledge developed during imitation training as described in the following section.

### Adult Speech Processing Within Our Model Sketch

The adult model of speech processing (production and perception) can be separated in a linguistic-cognitive part and in a sensorimotor part. Moreover, the speech processing model comprises a *production pathway* and a *perception pathway*, but both pathways access the same knowledge repositories, i.e., the mental lexicon and the mental syllabary. The cognitive-linguistic part of the speech production network starts with cognitive processing on the concept level (thinking, decision making, forming intentions, etc.) followed by concept, lemma, and phonological form activation. The associated neural activation patterns appear within the concept, lemma, and phonological form buffers which are closely connected to the mental lexicon. Thus, the cognitive-linguistic part of the speech processing network transforms an intended utterance (or just a word) into a phonological representation or phonological form (Figure 1C). This level is comparable to the phonological form level following phonological encoding and preceding syllabification and phonetic encoding in the Levelt approach (Levelt et al., 1999) and this level is comparable with the phonological content buffer exemplified in the GODIVA model (Bohland et al., 2010).

On the perception side the cognitive-linguistic part of the speech processing network allows comprehension, i.e., concept activation based on the activation of a phonological form. The activation of the phonological form results for each acoustic input, if this input has been processed (or perceived) auditorily, i.e., after passing the sensorimotor part of the network (Figure 1C). In the context of the dual route approach of speech processing (Hickok and Poeppel, 2007, 2016) the level of phonological representation of perceived speech items follows the spectro-temporal signal processing module and precedes processing within the lexical and combinatorial network part of the ventral path.

On the production pathway side, the processing within the sensorimotor part of the production network starts with syllabification, i.e., with a fragmentation of the phonological sound sequence in chunks, which potentially can be directly executed as motor programs. Syllabification leads to an activation of motor plans, i.e., by activating *raw* gesture score for syllable-sized chunks as part of the incoming phoneme sequence (phonological form in Figure 1C). These raw gesture scores or discrete motor plan specifications are carrying not more information than a (segmental) phonological description, i.e., the phoneme sequence of the syllable itself (see below: concept of speech gestures). If a motor program exists for the syllable under production, this information is forwarded from the mental syllabary to the motor plan level (red arrow in Figure 1C) and the motor program of the syllable can be activated directly and subsequently the syllable can be executed. A motor program exists if that syllable has been trained during the imitation phase. If the syllable does not exist, it needs to be programmed in detail which starts with a *full specification* of the gesture score. In our model we have implemented two routes for realizing that process. (i) *Adapting approach*: The motor plan of a phonologically similar syllable can be activated, for which a motor program exists, and many quantitative parameters of the gesture score can be copied for a first version of the fully specified motor plan of the new syllable. This full specification affects quantitative parameters like duration of gestures and exact temporal coordination of beginning and ending of gestures while qualitative discrete (or phonological) gesture parameters are already set within the raw gesture score. (ii) *Assembling approach*: If no phonologically similar syllable exists, e.g., in case of the production of a CCV-syllable if only CV-syllables are acquired so far, the syllable can be fragmented in sub-syllabic parts like single consonants or CV-units like C@ (@ is SAMPA notation for schwa-sound) are activated and need to be assembled in order to build up a first fully specified motor plan which subsequently allows the generation of a first version of a motor program for the new syllable. An example is the generation of a motor plan for /pla/, which may be assembled from CV-syllables like /pa/ and /la/ or like /p@/ and /la/. This complex process is already established during the imitation phase of speech acquisition if more complex syllables need to be learned.

The task of fragmentation of the phonological sound chain of the utterance to be produced is called *motor planning*. Following Levelt et al. (1999) as well as Bohland et al. (2010), syllables are assumed to be to be basic units for *motor programming* and thus the phonological phase of motor planning is syllabification. Thus, the major task of motor planning is to identify syllabic units within the flow or sequence of phonological sounds already activated by the cognitive-linguistic part of the model. If the motor program exists for a syllable, the step of motor programming is just to activate and execute the motor program. If the motor plan does not exist, the planning needs to be extended by selecting sub-syllabic units and the subsequent process of motor planning is a complex procedure of combining sub-syllabic units.

In our model sketch (Figure 1C) the motor programs of already learned syllables are stored in combination with their appropriate sensory states (auditory and somatosensory states) in the mental syllabary. This is comparable to the fact that in GODIVA (Bohland et al., 2010) already existing (prelearned) motor programs are stored in the speech sound map.

A *bottom–up process for forwarding motor information* is introduced in our approach, i.e., forwarding the information, whether a motor program for a syllable exists or not from the level of the mental syllabary to the motor plan level (red arrow in Figure 1C) in order to allow the choice between direct motor program activation and motor planning.

A concrete neurobiologically inspired realization of specific parts of our sketch of a production model introduced here is given in section “Experiments” of this paper by introducing two different quantitative computer-implemented model approaches, which were used for the simulation of speech acquisition and adult speech production.

### Phonological Knowledge and Structural Specifications of Syllables

Phonological and phonotactic knowledge is important for successful motor planning. It is needed for dividing the phonological sound sequence in syllables as well as for selecting phonologically similar syllables in the case of motor programming of new syllables. Thus, the typical phonological representation of a syllable is its phoneme sequence, e.g., /ba/, /da/, /dat/, /bla/, /blat/, /pa/, /ta/ etc. As already reported above it can be assumed that adult speakers have knowledge concerning different types of syllables, i.e., concerning basic syllable structures like CV, CVC, CCV, CCVC, etc. With respect to phonological features the type of syllable can be specified in more detail, e.g., as BV, PV, NV, LV, BLV, BNV, etc. Here CV syllables are separated concerning its initial consonant, i.e., voiced vs. unvoiced plosive, and nasal vs. lateral (B, voiced plosives; P, voiceless plosives; N, nasals; L, laterals). Consequently, CCV syllables can be separated with respect to initial voiced plosive-lateral-consonant clusters, initial voiced plosive-nasal clusters and so forth. In the next section it will be shown that a phonological representation of a syllable is comparable with a raw specification of a gesture score. A concrete example for the realization (or implementation) of phonological knowledge is given in Supplementary Appendix A for the computer-implemented neural simulation model 2.

### The Concept of Speech Gestures and Gesture Scores

Gestures are target-directed dynamically defined movement units of speech articulation (Saltzman and Munhall, 1989; Browman and Goldstein, 1992; Goldstein et al., 2006; Kröger and Bekolay, 2019). Gesture scores define the temporal organization of gestures of a speech item like a word or a syllable. In the strict interpretation of Articulatory Phonology gestures and their temporal coordination are already defined at the lexical level for words. In our approach two levels of gestural representation are introduced. At the phonological level gestures are specified *discretely* (as feature bundles: *raw gesture score; discrete phonological specification of a motor plan*). At the sensorimotor level gestures are parameterized *quantitatively* by specifying the exact beginning and ending of each gesture activation within a gesture score, by specifying the (relative) articulatory velocity for reaching a target, and by specifying the exact target location. This results in a *phonetic or full specification of a motor plan*. This quantitative description of all gestures within a gesture score serves as basis for the generation of a detailed and complete neural activation pattern of all neuromuscular units controlling all articulators during the production of a speech item (*motor program*).

If a gesture is activated, it aims to reach a certain articulatory target in a certain time interval. Consonantal targets are places of articulation or location of constriction, as defined by features like labial, apical, or dorsal. Vocalic targets are specific tongue positions or specific vocal tract shapes, as defined by features like high, low, front, back, rounded, and unrounded. In the case of consonantal gestures, the definition of the gesture target also includes the definition of degree and type of constriction like full closure (plosives and nasals), near closure (fricatives), lateral closure (laterals), etc. The differentiation of plosives and nasals is achieved by introducing two further gestures, which are the velopharyngeal closing or opening gestures. Moreover, glottal opening and closing gestures appear for differentiating voiceless and voiced speech sounds. Thus, in the case of the velum and of the glottis, the goal of the gesture is the formation of a closure or of an opening of the glottal or velopharyngeal passage. Beside closing for phonation in case of the glottis (glottal closing gesture), a glottal tight closing gesture exists if a glottal stop sound needs to be produced. Beside closing for producing oral sounds in the case of the velum (velopharyngeal closing gesture), a velopharyngeal tight closing gesture needs to be activated simultaneously with the oral closure or near closure in case of plosives and fricatives. That guarantees an air-tight closure of the velopharyngeal port in case of obstruents (fricatives and plosives) for building up an oral pressure which is needed for producing frication noise in case of fricatives, or for producing a noise burst in case of plosives.

Gestures can be described as bundles of features, where the features mainly describe the gesture targets. It is shown below how single speech sounds (phonemes) can be built-up by one, two, or more gestures (see Tables 1, 2), even if the gesture is seen as a non-segmental unit in the framework of Articulatory Phonology (Browman and Goldstein, 1992). It should be mentioned that some gestures may only represent one single distinctive feature, e.g., velopharyngeal opening/closing gesture for nasal/oral or glottal opening/closing gesture for voiced/unvoiced, while other gestures determine more than one feature, e.g., vocal tract shaping gestures determine the features high-low and front-back; consonantal constriction forming gestures generally determine place and manner of articulation.

**TABLE 1:** On the relationship between phonemes and gestures.

Segment (phoneme)	Gestures, building up a segment (realizing that phoneme)
vowels (a, i, u, …)	vocal tract form gesture + labial form gesture + velopharyngeal closing gesture + glottal closing gesture
plosives, voiced	full closing gesture + velopharyngeal tight closing gesture + glottal closing gesture
plosives, unvoiced	full closing gesture + velopharyngeal tight closing gesture + glottal opening gesture
fricatives, voiced	near closing gesture + velopharyngeal tight closing gesture + glottal closing gesture
fricatives, unvoiced	near closing gesture + velopharyngeal tight closing gesture + glottal opening gesture
nasals	full closing gesture + velopharyngeal opening gesture + glottal closing gesture
lateral	lateral constriction gesture + velopharyngeal closing gesture + glottal closing gesture

**TABLE 2:** On the relationship between gestures and features.

Gesture	Features, determined by the gesture
vocal tract shaping gesture (vocalic)	high-low, front-back
labial shaping gesture (vocalic)	rounded-unrounded
velopharyngeal closing vs. tight closing gesture	sonorant vs. obstruent
velopharyngeal (tight) closing vs. opening gesture	oral (non-nasal) vs. nasal
full vs. near closing gesture (consonantal)	plosive vs. fricative (or nasal vs. fricative)
full closing gesture (consonantal)	labial, apical, dorsal
labial constriction or closing gesture (consonantal)	bilabial, labiodental
apical constriction or closing gesture (consonantal)	dental, alveolar, postalveolar
dorsal constriction or closing gesture (consonantal)	palatal, velar
lateral constriction gesture (consonantal)	lateral, alveolar
phonation vs. glottal opening gesture	voiced vs. voiceless

**FIGURE 2:**
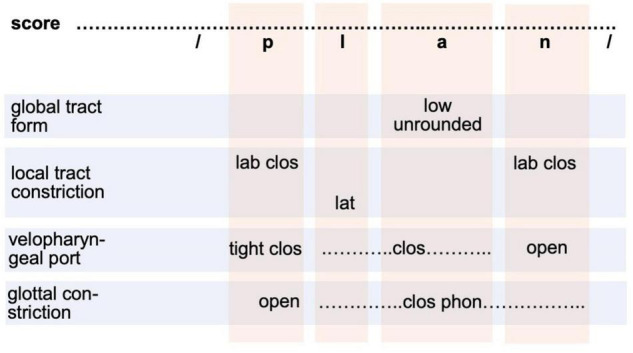
Raw gesture score (or motor plan specification in terms of the sketch model in section “The sketch for a model of speech production”) for syllable /plan/. Gestures are ordered on four articulatory tiers (rows as light blue boxes). Columns (light red boxes) indicate segment-gesture relations. The global tract forming gestures comprise two sub-gestures acting on different articulators: tongue and lips (see Table 2). The local constriction forming gestures act on three sub-tiers with respect to the gesture performing articulator: labial, apical, dorsal. Velopharyngeal and glottal gestures are listed in separate tiers. lab clos, labial full closing; lat, lateral; clos phon, closing for phonation.

In our adult production model (Figure 1C) the motor plan level is realized as raw gesture score. This specification directly results from the phoneme sequence (phonological form level in Figure 1C) but it simplifies the transition from a segmental-linguistic toward a motor description of each syllable. At the motor plan level, all gestures are specified and arranged in four basic tiers (light blue rows in Figure 2). These tiers represent primary articulators, i.e., the main organs with which gestures are performed. A bundle of gestures appears for each sound (see Figure 2 and Table 1). However, gestures assimilate if neighboring sounds show the same gestures on an articulatory tier (for example three neighboring sounds /lan/ are voiced in /plan/; see Figure 2). The vertical light red columns in Figure 2 indicate all gestures which are related to one sound.

**FIGURE 3:**
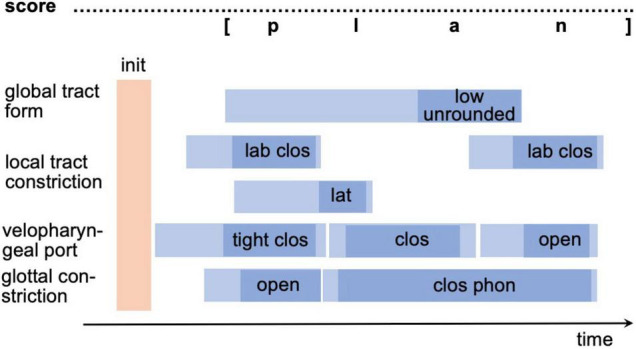
Exact temporal specification of gestures [or motor program specification in terms of the sketch model in section “The sketch for a model of speech production”] for syllable /plan/ in a gesture score (fully specified gesture score). For abbreviations see figure caption of Figure 2; init, time instant for motor program activation.

In order to generate a motor program from a motor plan (raw gesture score) all parameters of all gestures and the temporal coordination of all gestures need to be specified (fully specified gesture score). Thus, the exact points in time describing the beginning and the end of the neural activation of each gesture as well as describing the reaching and leaving of the spatial target region must be specified for each gesture. A full temporal specification of all gestures for a realization of /plan/ is shown in Figure 3. Light blue intervals in Figure 3 mark the time interval defining beginning and ending of neural activation for each gesture while the dark blue intervals mark the beginning and ending of the target phase of each gesture. Thus, the initial light blue time interval marks the target-directed movement phase and the final light blue time interval the release phase.

Beside the exact specification of temporal parameters and target parameters, motor programming needs information concerning the extent to which secondary articulators need to be involved in the execution of a gesture. Primary articulators are those mainly defining the gestures target (lips, tongue dorsum, tongue tip, velum, and glottis). A typical secondary articulator is the lower jaw in case of vocalic and consonantal gestures. For example, to implement the motor program of the syllable /ba/, it must be clear how much the lower jaw should be raised during the formation of the lip closure within the production interval of /b/ in order not to endanger the subsequent production of the /a/, because during the production of the /a/ the lower jaw must be lowered to a certain degree. Thus, conflicting requirements for the secondary articulators involved in gesture realizations of temporally neighboring gestures must be brought into harmony with one another, and consequently the displacement of the primary articulators relative to the secondary articulators must be adapted accordingly.

## Experiments

In this section, we describe two sets of simulation experiments (using two different neural modeling approaches, i.e., model 1 and model “The sketch for a model of speech production,” see below) that demonstrate key ideas described in the sketch of our overall model in Section “The sketch for a model of speech production.” A comprehensive implementation of the model sketch is reserved for future work. In simulation experiment 1 (using the computer-implemented model 1) babbling and imitation training is simulated for small vocabularies using a connectionist network approach including growing SOMs (Kröger and Cao, 2015; Kröger et al., 2019). In simulation experiment 2 (using the computer-implemented model 2) adult production for already learned as well as for new syllables is simulated using a spiking neuron network approach (Kröger et al., 2020). The computer-implemented babbling, imitation and adult production models used in these experiments are realizations of parts of the model sketch described above. Both computer-implemented models use different neuro-computational approaches. Model 1 implements nodes representing neuron ensembles and edges representing neural connections between nodes. Neural activity is averaged over defined time intervals as well as over neuron ensembles (see Kröger and Bekolay, 2019, p.133ff). This approach can be labeled as spatio-temporal activity averaging connectionist approach, while the spiking neuron approach used in model 2 (Eliasmith, 2013; Stewart and Eliasmith, 2014; Bekolay et al., 2014) includes the modeling of spatial and temporal details of neural processes and the modeling of neural control and decision processes.

### Experiment 1

The simulation of babbling and imitation training was done for ten virtual learners or virtual speakers, modeled by ten instances of our connectionist neural model of speech learning (simulation model 1, see Kröger et al., 2019). The main goal of this simulation experiment is to show how phonological knowledge can be gained during early phases of speech acquisition based on motor information and sensory information resulting from processing of sensory feedback. The architecture of simulation model 1 represents the babbling and imitation stage of the model sketch (see Figure 1A for babbling and Figure 1B for imitation). The phonetic map and the semantic map are implemented here in form of growing self-organizing maps (G-SOMs; Cao et al., 2014).

Self-organizing maps are able to represent the main features of a set of training items (Kröger and Bekolay, 2019). The network in which a SOM is included always comprises one or more state maps in which cognitive, motor or sensory states of training items can be activated, while the SOM itself represents the learned knowledge in a structured way. All neurons of each state map are connected with all neurons of the SOM. The state maps can be seen as an input–output interface within the neural network. The learning algorithm of SOM is shaped in a way that with increasing training by applying each training stimulus several times, each stimulus is represented in a specific local area (i.e., by a specific set of neurons) of the SOM. Different regions within a SOM represent different types of training items, or in other words, specific regions of a self-organizing neural map represent different features of items. Thus, SOM are often also labeled as feature maps.

A typical disadvantage of SOMs is the fact that the number of neurons building up this map needs to be defined in advance. In order to model the learning procedure of SOMs in a more natural way, a self-organizing neural map should grow during learning (i.e., should capture neighboring neurons so far not part of the network). Our G-SOM approach includes this demand by starting with a basic set of just 4 nodes, which allows a representation of one or two training stimuli, but in the case of applying more stimuli to the network, a driving force can be defined which leads to a recruitment of more nodes (neuron ensembles) in order to be able to represent the whole set of incoming stimuli within this growing SOM (GSOM, see Cao et al., 2014). After training, the growing self-organizing network including the growing SOM, all state maps, and all edges between the nodes of these maps can be driven in a way that an activation of a neuron within this network leads to an activation of each specific (generalized) state which is included in the training set. In the case of our model the activation of a node within the growing SOM leads to an activation of the motor and sensory states of all (generalized) speech items represented by the training set.

Babbling training starts with a set of 70 items which combine proto-consonantal labial, apical, or dorsal closing gestures with proto-vocalic gestures. At the beginning of babbling training, gesture targets varied freely with respect to degree and location of constriction. During babbling training, bidirectional neural connections are established between the phonetic map and the motor and sensory state maps in order to associate motor and sensory states (see section “Method”).

During imitation training bidirectional neural connections are established in addition between phonetic map (mental syllabary) and semantic map in order to associate motor and sensory states with concept states (meanings). The specification of neural connections as well as the adding of new nodes to both GSOMs is described in detail by Cao et al. (2014). The training corpus comprises 70 syllables (CV- an CCV-syllables). Each of these syllables are associated with a meaning thus establishing a word (Kröger and Cao, 2015). Five different vowels [V = /i, e, a, o, u/ and three different types of consonants; six plosives C = /b, d, g, p, t, k/, one glottal stop C = /?/(see the V-syllables in Kröger and Cao, 2015), two nasals C = /m, n/and one lateral C = /l/; 10 consonants in total] were allowed to be combined with each other resulting in 5 vowels × 10 consonants = 50 CV-syllables. Furthermore, four CC-clusters /bl, gl, pl, kl/ were allowed to be combined with all vowels resulting in 5 vowels × 4 CC-clusters = 20 CCV-syllables.

#### Method

In this connectionist model, concepts (Figure 1B) were represented by semantic feature bundles comprising 470 features (Cao et al., 2014). Thus, the neural representation of concept states comprises a neural state map of 470 neurons representing semantic features like “is living,” “can bark,” etc. The auditory state map comprises 24 × 64 neurons (nodes), where 24 neurons represent the frequency scale (bark scaled center frequencies) and 64 neurons represent a time scale (time steps of 10 ms; Kröger et al., 2019). The somatosensory state map comprises 4 × 64 neurons, where 4 neurons represent relative articulator to articulator distance for lips, articulator to vocal tract wall distance for tongue tip and tongue dorsum and a relative displacement value for the lower jaw (ibid.). The motor plan state map comprises 10 neurons for each gesture representing all gesture parameters (four points in time, two target values, one parameter naming the articulator) and the motor program state comprises 2 × 64 neurons representing the agonist/antagonist neuromuscular activation of each of the 10 model muscle groups (six model muscle groups for controlling lips, tongue tip, and tongue body; three model muscle groups for controlling velum, glottis, and lower jaw). The articulatory-acoustic model used was developed by Birkholz et al. (2011).

All syllables or words (concept, sensory, and motor states) are coded by *distributed neural representations* within the state maps. Here many neurons of each state map can be activated in parallel for representing a specific state. All syllables or words are represented locally by one neuron in each GSOMs (*local neural representation*). Here each neuron or node represents a learned word or syllable. The link weights of the neural connections between a specific GSOM node and all nodes of a state map directly represent the neural activation pattern for that state for a specific word or syllable.

Babbling trainings was carried out using an early version of our GSOM model and a set of proto-V and proto-CV training items as introduced by Kröger et al. (2009). A later imitation training was carried out using 210 training items item as introduced by Kröger and Cao (2015). Here, each of the 70 syllables was imitated or resynthesized three times. The resynthesis procedure was done manually (Bauer et al., 2009). Ten runs of imitation training were executed leading to 10 different training results, representing 10 instances of the model (10 virtual learners). Each run comprised 50 imitation training cycles with 1470 training steps per cycle, i.e., 7 training steps for each of the 210 training items per training cycle (Kröger et al., 2019).

#### Results

Babbling training results in an association of auditory and motor states with an error rate of less than 5% after 10 training cycles per babbling item (10 cycles × 1470 training steps). During later imitation training syllable-to-meaning associations were established after 50 training cycles (50 cycles × 1470 training steps) for 66 ± 3 words (whole corpus is 70 words). This leads to a mean error rate of about 5.7% for word production (in this case a node of the phonetic map represents two different words, Kröger et al., 2019). Production errors occur here because the model represents the state of speech experience of children of one to one and a half year. For all correct syllable-to-meaning associations the phonetic representation is reliable because all phonetic realizations of a syllable (all nodes representing a syllable in the mental syllabary) are coded by nodes which are in a direct neighborhood with a maxim distance of 2 intermediate nodes. This reflects the fact that after training phonetic realizations of the same word vary only in a small range.

**FIGURE 4:**
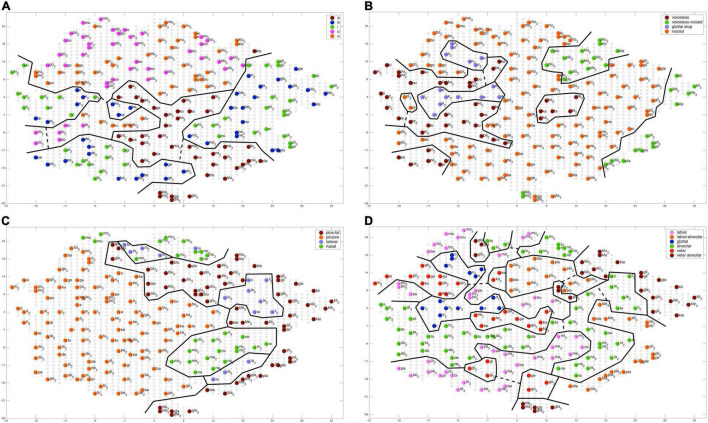
Topology of a self-organizing phonetic feature map (G-SOM) after 50 training cycles for training one of 10 training runs. Network nodes are labeled with respect to different features: **(A)** vocalic features; **(B)** voicing features; **(C)** manner of articulation for initial consonants; **(D)** place of articulation for initial consonants. Solid black lines indicate the borders of feature regions, dashed black lines indicate interconnecting pathways for sub-regions representing the same feature (see text). Outliers are not marked here. They appear within a specific feature region as nodes with a different color, i.e., with a color that differs from the main color of a feature region. The main color of a feature region indicates the feature represented by that region.

An evaluation of the number of feature regions per feature are summarized for each of 10 trained instances of the model (Table 3). A feature region is defined as a space within the G-SOM of the mental syllabary which includes all syllable nodes which represent syllables, which share at least one identical segmental feature value for type of vowel, for voicing, for place of articulation, and for manner of articulation (see Figure 4). Respecting the fact that our syllable corpus comprises two types of syllables (CV and CCV) this leads to a separation of (i) three vocalic features (vowel V within CV or CCV is a front vowel /i/ or /e/, vowel is a back vowel /o/ or /u/, vowel is the low vowel /a/), (ii) four glottal features (initial C of a CV syllable is voiced or voiceless, initial C of CV is voiceless, initial C of a CV is a glottal stop, both CC’s in the CC-cluster within a CCV are voiced, or CC-cluster within CCV is a voiceless consonant followed by a voiced consonant), (iii) four consonantal features specifying manner of articulation (initial C in CV is a plosive, a lateral, or a nasal; the initial CC-cluster in CCV is a plosive followed by a lateral) and (iv) six consonantal features specifying the place of articulation (place of articulation of initial C in CV is labial, alveolar, velar, or glottal, place of articulation in the CC-cluster of CCV-syllables is labial for the first and alveolar for the second consonant or velar for the first and alveolar for the second consonant).

**TABLE 3:** Number of feature regions for each of 10 trained model instances (tr01, …, tr10) and median for all single features as well as for feature groups.

		tr01	tr02	tr03	tr04	tr05	tr06	tr07	tr08	tr09	tr10	Median: single features	Median: feature group
vocalic features	front	1	1	2	1	1	1	2	1	1	1	1	1
	back	1	1	1	1	2	1	1	1	2	1	1	
	low	1	1	1	1	1	1	1	2	1	1	1	
glottal features	voiced	1	2	1	1	1	1	1	2	3	1	1	2
	v.less	2	3	2	2	2	3	2	1	3	2	2	
	glott.stop	3	3	1	2	1	2	3	3	3	1	2,5	
	v.less-voi	2	1	1	2	1	3	3	3	2	2	2	
consonantal feature: manner	plosive	2	1	1	1	2	1	1	1	1	1	1	2
	lateral	2	2	3	4	3	3	3	2	3	3	3	
	nasal	2	3	2	3	1	3	4	2	1	2	2	
	plos-lat	1	1	1	2	1	2	3	2	2	1	1,5	
consonantal feature: place	labial	3	4	2	3	3	3	4	1	2	2	3	2
	alveolar	1	2	3	1	2	1	4	4	3	2	2	
	velar	3	3	3	3	2	3	1	2	3	2	3	
	glottal	3	3	1	3	1	2	2	3	2	1	2	
	lab-alveo	2	2	3	3	1	2	4	2	2	2	2	
	vel-alveo	2	2	3	1	1	3	3	2	3	3	2,5	
type of syllable	CV	1	2	1	1	1	1	1	1	1	1	1	1
	CCV	1	1	1	2	1	2	3	2	2	1	1,5	

*Sub-regions are counted as one feature region and outlier-regions are not included (see text).*

Feature regions (regions bordered by solid lines in Figure 4) were extracted manually here by applying the following rules: (1) A (sub-)feature region only includes nodes which are associated with syllables which are related to this feature. Moreover, all nodes need to be in direct neighborhood. (2) Two sub-feature regions are labeled as one feature region if they are in a relative neighborhood. That is the case if all three conditions stated below are fulfilled: (a) nearness: an interconnection of a length of less than 10 (free) network nodes can be found; (b) coherence: the interconnecting pathway does not cross more than one other feature region; (c) neutrality: network nodes representing a speech item (i.e., occupied network nodes) are not allowed to appear within this pathway. Interconnections between subregions representing one feature are indicated by dashed lines in Figure 4. (3) Outlier region: A subregion is not included in our evaluation if it appears with only one node.

The median of the sum of feature regions per feature is calculated for all single features as well as for all features belonging to a feature group for each of the ten model instances. “Type of syllable” is listed in Table 3 as well because it reflects an important phonotactic feature (CV vs. CCV). The median over all model instances for all features and all feature groups is low (≤3). This reflects a high degree of ordering of items with respect to all features at the level of the mental syllabary.

It can be concluded that our G-SOM realizations for the mental syllabary are capable to separate and thus to represent all features for all feature groups in an organized manner that is reflective of basic neural topography and map formation observed across multiple model instances. This can be seen as an indicator for the fact that the model is able to abstract these features and feature groups for describing phonological contrast if babbling training is done. It can be assumed that the model now is capable to establish a level of phonological representation as indicated in Figure 1C.

### Experiment 2

In Experiment 2 the adult production of monosyllabic words is simulated for already learned and for new words respectively new syllables. It is demonstrated that single word production can be successfully simulated using a spiking neuron model (simulation model 2). This holds for already learned words as well as for new words and their corresponding syllables. In case of new syllables, the process of activating phonologically similar syllables for adapting motor program information (see “adapting approach,” section “Adult speech processing within our model sketch”) is described in detail here.

**FIGURE 5:**
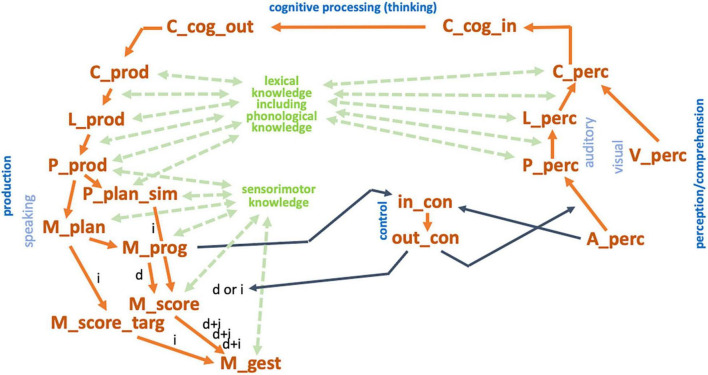
Architecture of the computer-implemented model 2 used for simulation Experiment 2. Yellow-ocher: Neural buffers of perception pathway, production pathway, control module, and input and output buffers for cognitive processing (C_cog_in/C_cog_out: cognitive input/output buffer (concept level); in_con/out_con: input/output buffer of control module; …_prod/…_perc: buffers within the production/perception pathway with: C_…/L_…/P_… representing the concept, lemma, or phonological level and with A_…/V_…/M_… representing the level of auditory, visual, or motor representations; see also Supplementary Appendix D). In this experiment, cognitive processing is not modeled (only a direct forwarding the perceived speech input into the production pathway). Yellow-ocher arrows indicate the associative buffers which realize the neural associations between specific neural buffers (see also Kröger et al., 2020). Green: Knowledge reservoirs for lexical including phonological knowledge and for sensorimotor knowledge. Knowledge is implemented by semantic pointer networks (for S-pointer networks see Kröger et al., 2020). These networks determine similarity relations between semantic pointers representing concepts, lemmata, phonological forms, and motor plans. Moreover, these networks determine the relationships between concepts and lemmata, lemmata and phonological forms, phonological forms and motor plan, motor plans and motor programs or gesture scores, and between gesture scores and their appertaining gestures. Black arrows indicate input and output information for the control module of the neural network. The auditory input information from the perception side is used for starting the production process. The motor program input from the production side (buffer M_prog) indicates, whether a motor program is already stored in the mental syllabary or not. This information allows the control module (i) to start the production of the word or syllable (from buffer A_perc) and (ii) do decide whether the direct or indirect route (d or i) needs to be taken for syllable production. Direct route (d): If the motor program exists, an activation occurs in buffer M_prog) which leads to a further direct activation of the syllable oscillator (buffer M_score) and to the activation of the gestures associated with that syllable (buffer M_gest). Indirect route (i) also called programming route (assembling or adapting syllables): If the control model gets the information that for a currently activated motor plan (buffer M_plan) the appertaining motor program does not exist (no neural activity in buffer M_prog) and the indirect route is activated by the control module: activation of motor plan (buffer M_plan) leads to activation of a similar motor plan in buffer P_plan_sim. This leads to a co-activation of the syllable oscillator for that similar syllable (from buffer P_plan_sim to buffer M_score) and to an activation of the correct gestures (from buffer M_plan to buffer M_score_targ). This leads to a first estimation of the gesture score for the planned syllable which is activated subsequently in M_gest. This gesture score is based on gesture timing from the similar syllable, taken from M_score, but it includes the correct gestures as demanded in the motor plan, which are adapted from M_score_targ.

The model used here (simulation model 2) is based on a spiking neuron approach including a detailed modeling of time-dependent neural processes by using the Neural Engineering Framework including the Semantic Pointer Architecture (Eliasmith et al., 2012; Eliasmith, 2013; Stewart and Eliasmith, 2014). The architecture of simulation model 2 represents the adult speech production which is part of the model sketch (see section “The sketch for a model of speech production” and see Figure 1C). The cognitive linguistic component comprises concept, lemma, and phonological form level (Figure 1C and see also Figure 5; Kröger et al., 2020). Semantic similarities of concepts as well as phonological similarities of syllables were modeled here using semantic pointer networks (Crawford et al., 2015; Kröger et al., 2016, 2020). In this approach semantic pointers represent meaningful neural activity patterns of words, lemmas, phonological forms of syllables (segmental phonological description of syllables, e.g., /plan/), or motor plan forms (cf. Figure 2) which can be activated in neural buffers. Modeling gestures and their temporal coordination (cf. Figure 3) is realized here using neural oscillators which are implemented as neuron ensembles (Kröger et al., 2021). A model language of 45 monosyllabic words (CV- and CCV-syllables, see Supplementary Appendix B) including an arbitrary mapping of word meanings to the phonological representation of theses syllables has already been learned and coded as sets of semantic pointers (lexical and phonological knowledge repository, see Figure 5).

Neural activation patterns of phonological forms appear within the phonological buffers P_prod or P_perc (for abbreviations of neural buffers see legend of Figure 5 and Supplementary Appendix D). The related semantic pointer network for phonological forms comprises all four layers of phonological representations (see last paragraph of this section) which are implemented as deep layers in the S-pointer network of phonological forms (for S-pointer networks and deep layers see Kröger et al., 2020 and Supplementary Appendix A).

**FIGURE 6:**
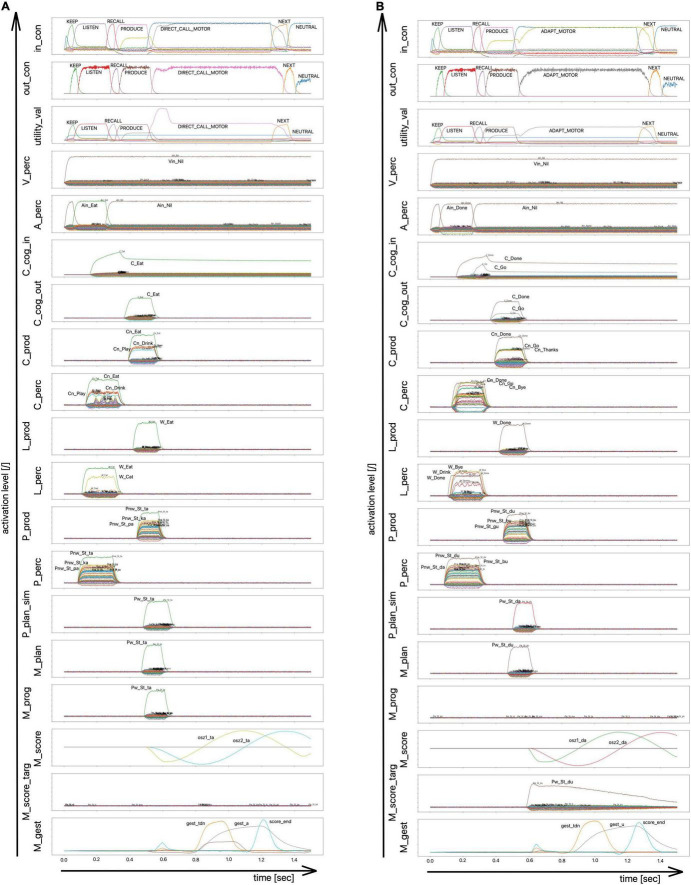
Neural activation patterns as function of time for different buffers of simulation model 2 for two different word (syllable) productions. (i) Word production task for the word “eat” [left side: **(A)** the word has been trained] and (ii) for the word “done” [right side; **(B)** the word has not been learned yet]. The architecture of simulation model 2 is given in Figure 5 and all acronyms for buffers are explained in Supplementary Appendix D. The ordering of buffers in this figure reflects the hierarchy i.e., the ordering of levels of the simulation model (cf. Figure 5): control module (con_in, con_out, utility_val) and input signals (A_perc, V_perc), cognitive processing module (C_cog_in, C_cog_out), perceptual and productive access buffers to the mental lexicon’s cognitive level (C_perc, C_prod), to its lemma level (W_perc, W_prod), and to its phonological form level (P_perc, P_prod), followed by planning buffers (P_plan_sim, M_plan), programming buffers (M_prog, M_score, M_score_targ), and execution buffer (M_gest).

Figure 6 shows neural activation patterns for different neural buffers and neuron ensembles as function of time for the simulation of word production for the word “eat,” represented arbitrarily by the syllable /ta/(already learned; motor program available) and for the simulation of the word “done,” represented arbitrarily by syllable/du/ (not yet learned; motor program does not exist; here a phonologically similar syllable needs to be activated and adapted as new motor program; for the arbitrary linking of meaning and phonological form of syllables see Supplementary Appendix B). The top two buffers in Figure 6 (in_con, out_con) represent incoming and outgoing control activity. The selection process for control actions results from comparing utility values (Figure 6, third row labeled utility_val) which are associated with different potential control actions. The control action used here is the selection of the direct or indirect production pathway (labeled as “d” or “i” in Figure 5). In case of an existing motor program for an already activated motor plan, the direct route is used (see Figure 5; the concept of direct and indirect pathways in motor planning/programming has been described as dual route theory by Whiteside and Varley, 1998; see also Miller and Guenther, 2021). In case of a non-existing motor program this program will be assembled or adapted from motor plan information of a similar syllable (indirect route).

The whole production activity, modeled in this Experiment 2 works as follows: The model (the speaker) starts with listening to auditory input and activates the target word which should be produced (control action LISTEN, Figure 6). The listening process uses the perception pathway of the model, i.e., subsequent activation of the target word within the buffers A_perc (auditory input level), P_perc (phonological level), and L_perc (Lemma level) and C_perc (concept level; see also Figure 5). The word is stored for a short time interval at the concept level (C_cog_in) but no other cognitive processing is done than forwarding the word toward the production pathway (via C_cog_out toward C_prod). Now the word passes the production pathway within the cognitive-linguistic part of the model via C_prod (concept level), L_prod (lemma level) toward P_prod (phonological level).

Subsequently, the production process activates the motor plan of the syllable (buffer M_plan; see Figures 5, 6). In case of the syllable/ta/(word “eat,” Figure 6A) a motor program exists and can be activated. In case of syllable/du/(word “done”) no motor program exists (no activity occurs in buffer M_prog; see Figure 6B) and the activation of a similar syllable (/da/) is further processed (from buffer P_plan_sim to buffer M_score and from buffer M_plan to M_score_targ; see Figures 5, 6B). In case of syllable /ta/ (Figure 6A) the motor program is directly executed via activation of the syllable oscillator M_score and subsequently the gestures associated with this syllable are activated (M_gest; here gest_tdn represent a consonantal gesture and gest_a represents the vocalic gesture; score_end informs the control component of the model that the next syllable can be activated; see also Supplementary Appendix C). In case of the syllable /du/ (Figure 6B) the motor program of a phonologically similar syllable is activated in buffer P_plan_sim (see activation of /da/in that buffer in Figure 6B) which triggers the activation of the syllable oscillator of the target syllable /da/. The semantic pointer which is now activated within the M_score_targ buffer gives the information, which gestures needs to emerge in the motor program for /du/. Thus, the new syllable /du/ is programmed by using the temporal information from the fully specified gesture score (motor program) of /da/, and by substituting the target of the vocalic gesture from the /a/ -target to the /u/ -target.

The control actions DIRECT_CALL_MOTOR vs. ADAPT_MOTOR (control module, buffer con_out, see Figure 5) determine whether the information of buffer M_plan or of buffer P_plan_sim is used for selecting or adapting a motor program. No activity in buffer M_prog (i.e., motor plan does not exist) leads to activation of ADAPT_MOTOR (indirect route, see Figures 5, 6B) and subsequently leads to activation of M_score_targ (based on P_prod). Activity in buffer M_prog indicates the existence of the motor program for that syllable and subsequently leads to a direct activation of M_prog and M_score. Moreover, the control actions mentioned above determine whether the current motor program information (buffer M_prog) needs to be modified by taking into account the information from buffer M_score_targ (case of adaptation: ADAPT_MOTOR) or whether the current motor program of the currently activated syllable can be used directly (case direct route: DIRECT_CALL_MOTOR, see Figures 5, 6A).

The current version of simulation model 2 is capable of processing already learned words in the cognitive-linguistic part of the model. It is assumed that syllable learning by adapting an already learned similar syllable starts at the motor plan level using the processes within the sensorimotor component mentioned above in this section, because the phonological to concept relations for that new syllable are learned later, i.e., if the syllable can be produced already at the sensorimotor level. Furthermore, it can be assumed that this sensorimotor production process needs to be repeated a few times for that new syllable before the syllable becomes part of the mental syllabary and thus is available for direct motor plan execution. The current version of simulation model 2 is not able to model this learning process as well. Moreover, the association of the phonological form to the concept needs to be learned and stored as a new word entry in the mental lexicon as well. Thus, simulation model 2 only gives us a first impression how a *first production (or imitation) trial for a syllable adaptation process* can be spelled out in this spiking neuron-based modeling approach before this syllable is consolidated in the mental syllabary and the associated word is consolidated in the mental lexicon. In our current version of model 2 a temporary meaning-to-phonological form association is already available in the mental lexicon, but it would perhaps be more realistic to start the motor program adaptation process on the phonological form level of the syllable within the production pathway.

The vocabulary used in this simulation experiment covers 45 monosyllabic words and their associated syllables (see Supplementary Appendix B). The syllable corpus comprises 27 CV-syllables and 18 CCV-syllables. CV-syllables include all combinations of nine consonants, i.e., six plosives /b/, /d/, /g/(three voiced plosives) and /p/, /t/, /k/(three voiceless plosives), two nasals /m/ and /n/, one lateral sound /l/, and three vowels, /i/, /a/, and /u/. CCV-syllables include all combinations of six consonant clusters, /bl/, /gl/, /pl/, /kl/, /gn/, and/kn/, and of the three vowels.

Four different types of *phonological structure features* were differentiated in our model (see also Supplementary Appendix A): (i) type of *syllable* (values: CV, and CCV); (ii) type of *gesture score* (values: BV, PV, NV, and LV, for CV-syllables and PLV, BLV, PNV, and BNV for CCV-syllables; with B, voiced plosives; P, voiceless plosives; N, nasals; L, lateral); these types of gesture score can be seen as subtypes of syllables like BV vs. PV and are forming groups of nearly identical gesture scores with the same ordering and same types of gestures at a specific temporal position; (iii) type of *segments* within the syllable (values: /Ca/, /Ci/, /Cu/, /bV/, /dV/, /gV/, /pV/, /tV/, /kV/, /mV/, /nV/, and /lV/ for CV-syllables and /CCa/, /CCi/, /CCu/, /bCV/, /gCV/, /pCV/, /kCV/, /ClV/, and /CnV/ for CCV-syllables); (iv) type of a *feature* of a segment within the syllable (values: V_high, V_low, V_front, V_back, C_full, C_lat, C_lab, C_api, C_dors, C_nas, C_nonas, C_voice, and C_vless for CV-syllables, C1_lab, C1_dors, C2_lat, C2_nas, CC_nonas, C1_voice, and C1_vless for CCV-syllables, and V_high, V_low, V_front, and V_back for CV- and CCV-syllables). For understanding the meaning of each phonological structure feature and of its values, the values of all four types of phonological structure features are compared with each other in Table 4. These four different types of phonological structure features defining *four layers of phonological representations* are used below for defining *five different levels of phonological knowledge* (see section “Method”).

**TABLE 4:** Comparison of values of phonological structure features for all types of syllables occurring within the vocabulary.

Type of syllable	Features	Segments	Scores
CV	full closure	bV, dV, gV, pV, tV, kV, nV, mV	BV, PV, NV
CV	lateral	lV	LV
CV	labial	bV, pV, mV	–
CV	apical	dV, tV, nV, lV	–
CV	dorsal	gV, kV	–
CV	nasal	mV, nV	NV
CV	oral (non-nasal)	bV, dV, gV, pV, tV, kV, lV	BV, PV, LV
CV	voiced	bV, dV, gV, mV, nV, lV	BV, NV, LV
CV	voiceless	pV, tV, kV	PV
CV	high	Ci, Cu	–
CV	low	Ca	–
CV	front	Ci	–
CV	back	Cu	–
CCV	labial C1	blV, plV	–
CCV	dorsal C1	glV, klV, gnV, knV	–
CCV	lateral C2	blV, glV, plV, klV	BLV, PLV
CCV	nasal C2	gnV, knV	NV
CCV	oral (non-nasal)	blV, glV, plV, klV	BLV, PLV
CCV	voiced C1	blV, glV, gnV,	BLV, BNV
CCV	voiceless C1	plV, klV, knV	PLV, PNV
CCV	high	CCi, CCu	–
CCV	low	CCa	–
CCV	front	CCi	–
CCV	back	CCu	–

*An empty field in the case of scores indicates that this set of syllables is not represented by score values. This holds only for specifications of different places of articulation or types of vowels.*

Simulation experiments (see sections “Method,” “Results for experiment 2a: CV-syllable learning,” and “Results for experiment 2b: CCV-syllable learning”) were performed using different levels of phonological knowledge in model 2 in order to evaluate (i) how much phonological knowledge is needed in order to adapt new syllabic motor programs from the motor plan and motor program information of similar syllables, (ii) which layers of phonological representations are most relevant for detecting similar syllables in order to perform a successful adaptation process for new syllables, and (iii) how the amount of phonological similarity between a detected similar syllable and the intended new syllable (i.e., the amount of gesture targets which need to be adapted) depends on the different levels of phonological knowledge.

### Method

Ten different versions or variants of the production model (10 different “virtual speakers”) were trained with respect to a variation in two different categories. Category 1 are five different levels of phonological knowledge. These levels are (i) all types of phonological structure features are available, (ii) all types minus scores, (iii) all types minus segments, (iv) all types minus the segment features, and (v) all types minus scores and minus segment features are available. Category 2 are two different levels of the model concerning the state of speech acquisition, i.e., concerning the level of already learned syllables. These levels are: (i) CV-learning stage: all CV-syllables with V = /a/ are already learned: CV-syllables with V = /i, u/ and all CCV-syllables (V = /i, a, u/) have yet to be learned; (ii) CCV-learning stage: all CV-syllables are learned (V = /i, a, u/), and all CCV-syllables with V = /a/ are learned, but CCV-syllables with V = /i, u/ have yet to be learned.

In part one of the simulation experiment (simulation experiment 2a) only CV-syllables were trained based on the acquisition level (i). All five different levels of phonological knowledge were simulated for producing each CV-syllable three times. In this experiment (2a) 5 levels × 3 trials × 27 CV-syllables = 405 simulation trials were carried out. The simulation trials can be differentiated according to whether a word can be produced directly (motor program of corresponding syllable exists; this is the case for 9 of 27 syllables, i.e., for 135 simulations) or whether a word (respectively a syllable) has not yet been trained (motor program needs to be programmed; 18 of 27 syllables, i.e., 270 simulations).

In part two of the simulation experiment (simulation experiment 2b) only CCV-syllables were trained based on the acquisition level (ii). All five different levels of phonological knowledge were simulated. In this experiment (2b) 5 levels × 3 trials × 18 CCV-syllables = 270 simulation trials were carried out. The simulation trials can be differentiated according to whether a word can be produced (this is the case for 6 of 18 syllables, i.e., for 90 simulations) or whether a word (respectively syllable) has not yet been trained (12 of 18 syllables, i.e., for 180 simulations).

### Results for Experiment 2a: CV-Syllable Learning

**FIGURE 7:**
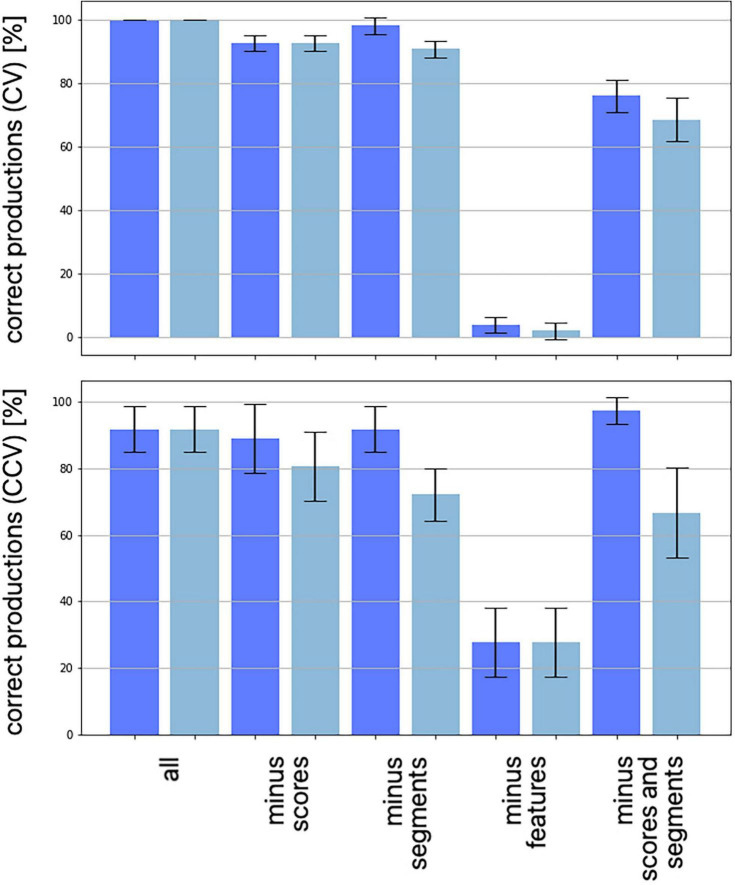
Percentage of correct productions for CV syllables and CCV syllables in case of five different degrees of phonological knowledge available during motor planning. These levels are (i) **all** four types of phonological structure features are available (type of *syllable*, type of *gesture score*, type of *segments* within the syllable, and type of a *feature* of a segment within the syllable; see section “Experiment 2” for a detailed description of all types of features and possible feature values), (ii) all types of structure features **minus scores** are available (only the phonological information concerning the structure feature “type of gesture score” is not available), (iii) all types **minus segments** are available (only the phonological information concerning the structure feature “type of segments within the syllable” is not available), (iv) all types **minus features** are available (only the phonological information concerning the structure feature “type of feature of a segment within the syllable” is not available), and (v) all types **minus scores and minus segments** are available (only the phonological information concerning the structure feature “type of gesture score” and concerning the structure feature “type of segment within the syllable” is not available). Furthermore, we separated the types of similar syllables with respect to the amount of similarity. Left side, dark blue: high degree of similarity: only the target of the vocalic gesture needs to be adapted. Right side, light blue: lower degree of similarity: target of the vocalic gesture and the target of up to two gestures affecting consonants within the syllable needs to be adapted.

In the case of the 135 simulations of producing already learned words (CV-syllables with V = /a/), no errors occurred (see Figure 7 top). Thus, already learned syllables can be easily produced in our model because the motor program of the corresponding syllable already exists. In the case of the remaining 270 simulations, depending on the type of phonological knowledge (five levels, see above), a phonologically similar motor program cannot be activated directly in several cases. For each model instance representing a specific type of phonological knowledge 3 trials × 18 CV-syllables = 54 simulations were performed for those CV-syllables for which no motor program exists (syllable has not yet been learned), i.e., for the CV-syllables with V = /i/ and V = /u/.

If all phonological structure features are available (case “all”; full phonological knowledge), the most similar syllable is activated directly in all cases of simulated production attempts. This means that the chosen most similar syllable always shows the same type of gesture score as is needed for adapting a specific new syllable and thus allows a successful adaptation process. The selected phonologically similar syllable differs only concerning the vowel target in this case.

In the case of the phonological knowledge level “all minus scores” no phonologically similar syllable can be activated at the first production attempt for 4 out of 54 cases, but correct similar syllables are activated in 92.6% of all attempts. In case of “all minus segments” that holds for only 1 out of 54 runs, leading to 98.1% correct productions, in case of “all minus scores and minus segments” that holds for 13 out of 54 runs (75.9% correct productions) but in case “all minus features” that holds for 51 out of 54 runs (only 5.6% correct productions; see Figure 7 top, left columns per knowledge level).

These results describe cases in which the difference between the chosen phonologically similar syllables and the syllable for which the motor program needs to be generated (new syllable) is up to two consonantal features beside the vocalic feature. If the degree of phonological similarity is thus high, that only the vocalic feature is different, i.e., all consonantal features are correct and thus no gesture needs to be adjusted but the vocalic gesture, the percentage of productions decreases by about 7.4% (4 runs) in case of “all minus segments” and in case of “all minus scores and minus segments,” and for about 1.8% (one run) in case of “all minus features” [see Figure 7 by comparing the left (dark blue) and right (light blue) of each pair of bars; the right represents productions without any adjustment of consonantal gestures; the left represents productions, in which up to two consonantal features need to be adjusted]. The percentage does not decrease in the case “all” and in case “minus scores.”

The most important result of Experiment 2a is that phonologically similar syllables for generating motor programs can be detected and activated in our model easily if the full phonological knowledge is available. Moreover, the strongest decrease for activating similar syllables occurs in case of reduction of phonological knowledge by “features.” In this case only 5.6% of all productions are correct, i.e., enable the activation of a phonologically similar syllable to start motor programming. The amount of similarity between similar syllable and new syllable (syllable under production) does not depend strongly in case of all different levels of phonological knowledge. Thus, in most cases of syllable adaptation the similar syllable differs only in one gesture parameter (as demonstrated in the example given in Figure 6B).

### Results for Experiment 2b: CCV-Syllable Learning

In the case of the 90 simulations of producing already learned words (CCV-syllables with V = /a/), no errors occurred. Learned syllables can therefore be easily produced in our model. In the case of the remaining 180 simulations, errors occurred (no phonologically similar syllable can be activated) in different quantity depending on the type of phonological knowledge available. For each type of phonological knowledge 3 trials × 12 CCV-syllables = 36 simulations were done for CCV-syllables with no motor programs available (syllables that had not yet been learned), i.e., for the CCV-syllables with V = /i/ and V = /u/.

If all phonological structure features are available (case “all”; full phonological knowledge), the most similar syllable for realizing the production of the new syllable is found directly for 33 out of 36 runs of simulated production attempts. Thus, in case of 91.7% of all productions a phonologically similar syllable is already found directly in the first run (Figure 7 bottom). Simulation results indicate, that in the remaining four runs, a phonologically similar syllable is found in the second production attempt, so that motor program generation here as well is possible without problems.

In the case of the phonological knowledge level “all minus scores” phonologically similar syllables can be activated directly in 32 out of 36 runs (88.9%), in case of “all minus segments” in 33 out of 36 runs (91.7%), and in case “all minus scores minus segments” in 35 out of 36 runs (97.2%). But in case “all minus features” the direct activation of a phonologically similar syllables occurs only in 10 out of 36 runs (27.8%; see Figure 7 bottom, left columns per knowledge level).

These results describe cases in which the difference between phonologically similar syllables and the syllable for which the motor program needs to be generated is up to two consonantal features beside the vocalic feature (mainly place or place and voice). If the degree of phonological similarity should be thus high, that only the vocalic feature is different, i.e., all consonantal features are correct, the percentage of productions decreases by about 8.3% (three runs) in case of “all minus scores,” decreases by about 19.4% (7 runs) in case of “all minus segments,” and decreases by about 30.6% (11 runs) in case of “all minus scores and minus segments.”

The most important result of simulation Experiment 2b is that in case of programming a new CCV-syllable a phonologically similar syllable can be detected and activated in 88.9% up to 97.2% of all trials depending on the level of phonological knowledge. Only in case of “all minus features” phonological knowledge is so small that a phonologically similar syllable is activated only in 27.8% of all production attempts. Thus, the results concerning the three points listed above [i.e., (i) how much phonological knowledge is needed in order to adapt new syllabic motor programs from the motor plan and motor program information of similar syllables, (ii) which layers of phonological representations are most relevant for detecting similar syllables in order to perform a successful adaptation process for new syllables, and (iii) how the amount of phonological similarity between detected similar syllable and intended new syllable depends on the different levels of phonological knowledge] are comparable for CV and CCV syllables.

## Discussion

A sketch for a model of speech production has been proposed including developmental aspects like the buildup of skills and speech knowledge during early phases of speech acquisition. While other models mainly concentrate on modeling of cognitive-linguistic aspects of speech production (e.g., Levelt et al., 1999) or mainly concentrate on modeling the sensorimotor aspects of speech production (e.g., Guenther, 2006; Bohland et al., 2010) it is the goal of our model sketch to give the complete view on speech production, i.e., linguistic as well as sensorimotor aspects. While the phonological level can be used for interfacing cognitive-linguistic and sensorimotor model parts of a speech production model in case of adult speech production the situation is more complex in early phases of speech acquisition. Thus, a comprehensive model of speech production needs to include the developmental processes occurring in speech processing. Our model sketch takes this into account by including early phases of speech acquisition, i.e., the babbling and the imitation phase.

While babbling constitutes a first realization of the sensorimotor part of the speech processing model, imitation establishes the cognitive-linguistic part and in addition further develops the sensorimotor part of the model. Imitation needs specific communication scenarios like triangulation (i.e., focusing an object and learning its meaning and its pronunciation by imitating the productions of the communication partner) and leads to the buildup of a mental lexicon as repository for concepts and lemmas as well as of the mental syllabary as a repository of sensory and motor forms of syllables. Here, imitation training tunes and differentiates already stored pre-linguistic babbling speech items (stored in a proto-syllabary, called phonetic map in our approach) into the direction of target-language specific speech items, mainly syllables. These assumptions play a central role in our model sketch and are based on literature (e.g., Levelt and Wheeldon, 1994; Oller, 2000; Cholin et al., 2006; Hickok et al., 2011; Buder et al., 2013; Lytle and Kuhl, 2017; Redford, 2019).

Furthermore, our sketch of a production model postulates that during the imitation phase the mapping between the items represented in the mental syllabary and in the mental lexicon introduces distinctiveness at the interface level between both repositories and thus converts phonetic into phonological features. This hypothesis is underlined by the emergence of phoneme regions at the level of the mental syllabary if the mental syllabary is modeled using a SOMs approach (e.g., Kröger and Cao, 2015). At the beginning of the imitation phase, phonological forms are not available which could be stored in the mental lexicon, but neural connections are established now between both repositories which associate words with syllables. Because the word-to-syllable association is established in a bidirectional way during the imitation phase (e.g., Kröger and Cao, 2015) firstly speech production can be simulated now by activating words to syllables from the mental lexicon toward the mental syllabary and secondly the dorsal stream of speech perception can now be simulated by using syllable-to-word associations from mental syllabary toward mental lexicon. Moreover, a successful word-to-syllable and syllable-to-word association allows the phonetic features to become categorical. Now, different feature values allow a separation of syllables which represent words of different meaning. In our model sketch a phonological level is established now, which on the side of speech production appears as interface between cognitive-lexical and sensorimotor processing and which on the side of speech perception now allows to establish the ventral stream of speech perception, which forwards speech items from the auditory processing via the phonological processing toward a lexical processing (cf. Hickok and Poeppel, 2007, 2016).

At the end of the imitation phase the adult speech processing model is established which comprises a cognitive-linguistic component as already introduced by Levelt et al. (1999) and a sensorimotor component which separates motor and sensory states and thus forward motor and feedback sensory processing (as introduced by Guenther, 2006; Bohland et al., 2010) and which separates motor planning and motor programming. In our model sketch, gesture scores are introduced as a vehicle for transforming segmental phonological syllable specifications into motor forms by specifying raw or categorical gesture scores followed by fully specified or quantitative gesture scores.

Two simulation experiments were carried out in this paper to substantiate distinct aspects of our sketch for a model of a speech production. In a first simulation experiment the model components are realized by implementing growing self-organizing networks for the sensorimotor as well as for the cognitive-lexical part of the model. A main result of this modeling is the ability of topographically organizing and later of differentiating speech items with respect to phonetic and later with respect to phonological features. Thus, the simulation of babbling and imitation by using growing SOMs exemplifies the emergence of phonological features based on knowledge gained from motor representations and sensory representations resulting from sensory feedback information.

In a second simulation experiment which is carried out by using a spiking neuron approach including an explicit modeling of time-dependent neural processes (Eliasmith, 2013) it is demonstrated how a new syllable is learned if motor programs for phonologically similar syllables are available. Here, the gesture timing parameters are copied from the already existing motor program of the similar syllable and only some gesture targets need to be exchanged to generate a first version of a motor program for the new syllable (adapting process). Further fine-tuning of gesture parameters may occur in further production attempts of this syllable. In two experiments it is shown that the phonological information concerning features like vocalic high-low front-back or consonantal place of articulation is important for allowing to select syllables exhibiting similar gesture scores. Moreover, it should be stated that phonological information can be used to specify or characterize segments as well as gestures at the motor plan level. What remains to be solved is the question how new *types* of syllables like first CCV-syllables can be learned if only CV-syllable motor plans are available (assembling process).

It is not the goal of the model sketch developed in this paper to combine segmental and gesture-phonological descriptions in one approach at each level of the model. As stated by Goldstein et al. (2006) segmental approaches in comparison to a gestural approach “appear to present problems … when they attempt to account for the temporal structure of speech - like regularities in relative timing between units, stochastic variability in that timing, and systematic variability in timing due to rate, speaking style, and prosodic context” (ibid., p. 222, footnote 6). Moreover, “temporal sliding of some (but not all) production units with respect to one another …” (ibid.) is not possible on the segmental level but increasing gestural overlap together with temporal reduction of duration of some gestures for example leads to significant effects at the segmental phonetic surface like assimilations and elisions as they appear in casual of fast speech. This has been demonstrated by Suprenant and Goldstein (1998) as well as by perceptual studies in early versions of our own gesture-phonological approach (Kröger, 1993). These results indicate that a gestural control approach cannot be replaced or mixed with a segmental control approach at a quantitative phonetic level where time and temporal relations between phonetic articulatory events come into play. And these facts are consistent with the sketch of a production model introduced in this paper. Within the sensorimotor part of our production model, we start with a raw gesture score description followed by a full quantitative specification of the gesture score for controlling articulation. A segmental phonological description of lexical units down to the syllable is introduced in our approach exclusively within the cognitive-linguistic model part.

Moreover, it is stated above that in our approach a raw gesture score which specifies gestures purely in a phonological manner as distinctive units can be converted into a segmental phonological description using phonemes as distinctive units and vice versa. Thus, our approach allows a description of lexical units by using a segmental or a raw gestural description comparable to that given by coupling graphs in the concept of Articulatory Phonology (Goldstein et al., 2006). But proto-syllables occurring in early phases of speech acquisition are described in our approach exclusively as gesture scores. Segmental phonological descriptions in our model appear later during speech acquisition and appear in our approach in the adult production model as a result of language-specific learning which occurs during the imitation phase. This is consistent with Goldstein et al. (2006, paragraph 7.2.3, p. 226): “What is the “glue” that allows articulatory gestures to be coordinated appropriately within a word form and that permits the establishment of lexically distinct coordination patterns across word forms? One possibility would be to hypothesize that gestures are organized into hierarchical segment and syllable structures that could serve as the scaffolding that holds the gestures in place through time. However, these relatively complex linguistic structures could only exist as part of an already developed phonology and could not be available pre-phonologically as part of an account of how articulatory gestures begin to be combined into larger structures.”

Our sketch of a model is based on well-known neurobiologically inspired approaches of speech production and speech perception (e.g., Levelt et al., 1999; Guenther, 2006; Hickok and Poeppel, 2007, 2016; Bohland et al., 2010; Guenther and Vladusich, 2012) and is consistent with these approaches. One further main goal of this paper was to highlight the importance of sensory feedback and thus to emphasize the importance of motor *and* sensory syllable representations at the level of mentally syllabary for establishing phonological knowledge and a phonological level during speech acquisition as interface between the cognitive-linguistic and the sensorimotor part of a production-perception model.

Moreover, a bottom–up pathway for motor information concerning already existing motor programs is introduced to enable the selection of separate processing routes for producing already learned syllables (direct route) versus producing syllables which are not learned so far and thus having no ready-made motor programs available (programming route). As part of the programming route the adapting process is implemented successfully and works satisfactorily if enough phonological information is available. Further work is needed for implementing the assembling process in order to generate motor programs for new types of syllables. This assembling process is not only an important process for adult speech production but also an important sub-process already occurring during the imitation phase of speech acquisition if new types of syllables must be acquired.

A limitation of our current modeling approach could be that the production of pseudowords is not included. But this reflects the fact that pseudoword production primarily appears in scenarios like logopedic diagnosis in case of suspicion on specific speech and language disorders or in case of suspicion of hearing loss. The main task in speech acquisition is that the child tries to communicate information (i.e., meanings in form of lexical items). Even if first production trials of words are relatively degraded it is the goal of the child to be understood by its caretaker or communication partner. The production of pseudowords differs from this goal but can be easily incorporated in our model sketch if a neural perception-production shortcut is included at the phonological form level as it has already been realized in our spiking neuron modeling approach for the simulation of phonological retrieval aids in case of an logopedic diagnostic word retrieval scenario (Kröger et al., 2020).

Both simulation experiments outlined in this paper can be seen as a proof of principle (i) for the idea how phonetic features – which appear in the sensorimotor representations of syllables at the level of the mental syllabary – become phonologically relevant by linking syllables with word meanings, (ii) how the emergence of knowledge and skill repositories (i.e., mental lexicon and mental syllabary) can be specified at the neural level as growth of neural maps and as an adjustment of neural connections between all neurons of these maps, (iii) how in case of speech perception and production of a word the flow and processing of information can be simulated in detail at concrete neural levels using a spiking neuron approach, and (iv) how specific processes of speech production like motor programming of a new syllable can be implemented in detail by adapting motor program features from phonologically similar and already learned syllables.

But in our current work we still must use two different neural modeling approaches in order to highlight distinct aspects of the model sketch. Model 1 (simulation experiment 1) is a comparably simple connectionist approach which is not capable of modeling spatial and temporal details like the generation of spike patterns (i.e., specific neural activation patterns for single neurons) but which allows the quantification of mean activation rates over specific time intervals (like activation interval for selecting a lexical item) and over a set of neurons (like neuron ensembles or neuron buffers representing a specific cognitive, lexical, sensory, or motor item). Model 2 (simulation Experiment 2) is a more detailed spiking neuron approach capable of modeling the spiking behavior of cortical neurons, which subsequently allows a detailed and straight forward modeling of the temporal aspects of the flow and of the processing of neural activation patterns within the speech production-perception network. It is a main goal or our future work to unify this modeling approaches into one (probably spiking neuron) approach capable of instantiating all developmental aspects and all processing aspects of the production-perception network. Currently one of the main difficulties is to model developmental aspects in a spiking neuron approach because of the immense computational loads appearing in learning scenarios.

## Data Availability Statement

The raw data supporting the conclusions of this article will be made available by the authors, without undue reservation.

## Author Contributions

BK developed the raw architecture of both simulation models (GSOM-model = model 1; Nengo-model = NEF-SPA model = model 2), conducted the experiments, and wrote the manuscript. TB developed main routines for simulation model 2 while MC developed main routines for simulation model 1. TB, MC, and BK together developed the detailed architecture of both simulation models.

## Conflict of Interest

TB is employed by Applied Brain Research. The remaining authors declare that the research was conducted in the absence of any commercial or financial relationships that could be construed as a potential conflict of interest.

## Publisher’s Note

All claims expressed in this article are solely those of the authors and do not necessarily represent those of their affiliated organizations, or those of the publisher, the editors and the reviewers. Any product that may be evaluated in this article, or claim that may be made by its manufacturer, is not guaranteed or endorsed by the publisher.
