# Supplementary material for: On the Emergence of Phonological Knowledge and on Motor Planning and Motor Programming in a Developmental Model of Speech Production
Source: Front Hum Neurosci. 2022 May 12;16:844529. doi: 10.3389/fnhum.2022.844529 (PMC9133537; doi:10.3389/fnhum.2022.844529)
Supplement: Supplementary file 1 [file Presentation_1.pdf]

Appendix A: S-pointer network for phonological representations

Four different types of phonological structure features were differentiated in model 2, i.e., type of syllable, type of gesture score, type of segments within a syllable, and type of feature of a segment within a syllable (section 3.2). These phonological structure features define similarity relations between syllables. The phonological form of syllables and their similarity relations are coded in S-Pointer networks. The definition of this S-pointer network comprises three components.

- A *syllable subnetwork* for the phonological forms (labeled as "phonos") contains S-Pointers for all CV- and CCV-syllables represented in our simulation model 2. These syllables are described here by their segmental phonological form (/ba/, /da/, /ga/ etc.).
- A *structure feature subnetwork* contains S-pointer for all values of all four types of phonological structure features (e.g. /C_a/ represents all CV-syllables with vovel /a/, /Cvoice_V/ represents all CV-syllables with a voiced initial consonant, etc.) The subnetwork is labeled as "phonos_deep").
- In addition, relation types are defined in order to specify each syllable in the syllable subnetwork by a set of structure feature values as defined in the structure feature subnetwork (e.g., "isScoreType" is a structure feature which defines a specific type of gesture score.

The description of relation types and S-pointer networks is done in the Nengo framework (www.nengo.ai) using Python (Bekolay et al. 2014). More information about S-pointers, S-pointer networks and their implementation in our simulation model 2, which uses the Neural Engineering Framework including the Semantic Pointer Architecture (Eliasmith et al. 2012, Eliasmith 2013, Stewart & Eliasmith 2014, Crawford et al. 2015) is given in Kröger et al. (2016).

A1 Definition of relation types within the phonological S-pointer network:

Relation types (relation type operators) are defined for the S-pointers within the phonological S-pointer network. *inclPhon* = *seg_x*: a syllable includes the segment x, *inclFeature* = *feat_x*: the consonantal or vocalic segments within the syllable include the feature x, *isSyllStruct* = *struct_x*: the syllable is shaped by segmental structure type x, *isCoreType* = *type_x*: The syllable is shaped by gesture structure type x. Python code for defining the relation types:

```
# define relation types:
rel_type_phono_keys = [inclPhon, inclFeature, isSyllStruct, isScoreType]
```

A2 Definition of the syllable subnetwork:

This subnetwork (labeled as phonos in source code) specifies a list of syllables. Here, Pnw_St_... is a prefix in order to specify an S-pointer describing a phonological mono-syllabic word representation (Pw) within a S-pointer network (n) as a stressed syllable (St). Each syllable is specified with respect to specific values of three phonological structure features (inclFeature, isSyllStruct, and isScoreType). The syllable subnetwork itself differentiates two segmental syllable types, i.e., CV- and CCV-syllables using the relation type inclPhon (see below). Python code for defining the syllable subnetwork:

```
phonos = {
        # CV:
        'Pnw_St_ba': [(isScoreType, 'Pn_St_CVbdg'),
                  (isSyllStruct, 'Pn_St_C_V'),
                  (isSyllStruct, 'Pn_St_C_a'),
                  (isSyllStruct, 'Pn_St_b_V'),
                  (inclFeature, 'Pn_St_C_Vlow'),
                  (inclFeature, 'Pn_St_Cfull_V'),
                  (inclFeature, 'Pn_St_Cnonas_V'),
                  (inclFeature, 'Pn_St_Cvoice_V'),
                  (inclFeature, 'Pn_St_Clab_V')],
        'Pnw_St_da': [(isScoreType, 'Pn_St_CVbdg'),
                  (isSyllStruct, 'Pn_St_C_V'),
                  (isSyllStruct, 'Pn_St_C_a'),
                  (isSyllStruct, 'Pn_St_d_V'),
                  (inclFeature, 'Pn_St_C_Vlow'),
                  (inclFeature, 'Pn_St_Cfull_V'),
                  (inclFeature, 'Pn_St_Cnonas_V'),
                  (inclFeature, 'Pn_St_Cvoice_V'),
                  (inclFeature, 'Pn_St_Capi_V')],
        'Pnw_St_ga': [(isScoreType, 'Pn_St_CVbdg'),
                  (isSyllStruct, 'Pn_St_C_V'),
                  (isSyllStruct, 'Pn_St_C_a'),
                  (isSyllStruct, 'Pn_St_g_V'),
                  (inclFeature, 'Pn_St_C_Vlow'),
                  (inclFeature, 'Pn_St_Cfull_V'),
                  (inclFeature, 'Pn_St_Cnonas_V'),
                  (inclFeature, 'Pn_St_Cvoice_V'),
                  (inclFeature, 'Pn_St_Cdors_V')],
…
        'Pnw_St_mi': [(isScoreType, 'Pn_St_CVmn'),
                  (isSyllStruct, 'Pn_St_C_V'),
                  (isSyllStruct, 'Pn_St_C_i'),
                  (isSyllStruct, 'Pn_St_m_V'),
                  (inclFeature, 'Pn_St_C_Vhigh'),
                  (inclFeature, 'Pn_St_C_Vfront'),
                  (inclFeature, 'Pn_St_Cfull_V'),
                  (inclFeature, 'Pn_St_Cnas_V'),
                  (inclFeature, 'Pn_St_Cvoice_V'),
                  (inclFeature, 'Pn_St_Clab_V')],
```

```
'Pnw_St_ni': [(isScoreType, 'Pn_St_CVmn'),
          (isSyllStruct, 'Pn_St_C_V'),
          (isSyllStruct, 'Pn_St_C_i'),
          (isSyllStruct, 'Pn_St_n_V'),
          (inclFeature, 'Pn_St_C_Vhigh'),
          (inclFeature, 'Pn_St_C_Vfront'),

          (inclFeature, 'Pn_St_Cfull_V'),
          (inclFeature, 'Pn_St_Cnas_V'),
          (inclFeature, 'Pn_St_Cvoice_V'),
          (inclFeature, 'Pn_St_Capi_V')],
'Pnw_St_li': [(isScoreType, 'Pn_St_CVl'),
          (isSyllStruct, 'Pn_St_C_V'),
          (isSyllStruct, 'Pn_St_C_i'),
          (isSyllStruct, 'Pn_St_l_V'),
          (inclFeature, 'Pn_St_C_Vhigh'),
          (inclFeature, 'Pn_St_C_Vfront'),
          (inclFeature, 'Pn_St_Clat_V'),
          (inclFeature, 'Pn_St_Cnonas_V'),
          (inclFeature, 'Pn_St_Cvoice_V'),
          (inclFeature, 'Pn_St_Capi_V')],
…
 # CCV:
 'Pnw_St_bla': [(inclPhon, 'Pn_St_ba'), (inclPhon, 'Pn_St_la')],
 'Pnw_St_gla': [(inclPhon, 'Pn_St_ga'), (inclPhon, 'Pn_St_la')],
…
 'Pnw_St_gni': [(inclPhon, 'Pn_St_gi'), (inclPhon, 'Pn_St_ni')],
 'Pnw_St_kni': [(inclPhon, 'Pn_St_ki'), (inclPhon, 'Pn_St_ni')],
…
 'Pnw_St_gnu': [(inclPhon, 'Pn_St_gu'), (inclPhon, 'Pn_St_nu')],
 'Pnw_St_knu': [(inclPhon, 'Pn_St_ku'), (inclPhon, 'Pn_St_nu')],
}
```

A3 Definition of the structure feature subnetwork:

This subnetwork (labeled phonos_deep in source code) specifies all possible values for all four phonological structure features inclFeature, isSyllStruct, isScoreType, and inclPhon. The feature inclPhon is used here for building up and for differentiating CCV syllables from CV syllables. Python code for defining the structure feature subnetwork:

```
phonos_deep = {
        # specification of type of gesture score (isScoreType)
        'Pn_St_CVbdg' : [],
        'Pn_St_CVptk' : [],
        'Pn_St_CVmn' : [],
        'Pn_St_CVl' : [],

        # specification of type of segmental syllable structure
        # (isSyllStruct)
        'Pn_St_C_a': [],
        'Pn_St_C_i': [],
        'Pn_St_C_u': [],
        'Pn_St_b_V': [],
        'Pn_St_d_V': [],
        'Pn_St_g_V': [],
        'Pn_St_p_V': [],
        'Pn_St_t_V': [],
        'Pn_St_k_V': [],
        'Pn_St_m_V': [],
        'Pn_St_n_V': [],
        'Pn_St_l_V': [],

        # specification of type of feature appearing in vocalic or consonantal
        # segment position (inclFeature)
        'Pn_St_C_Vlow': [],
        'Pn_St_C_Vhigh': [],
        'Pn_St_C_Vfront': [],
        'Pn_St_C_Vback': [],
        'Pn_St_Cfull_V': [],   # plos+nas_V
        'Pn_St_Clat_V': [],    # lat_V
        'Pn_St_Cnonas_V': [],  # plos+lat_V
        'Pn_St_Cnas_V': [],    # nas_V
        'Pn_St_Cvoice_V': [],  # voiPlos+nas+lat_V
        'Pn_St_Cvless_V': [],  # vlessPlos_V
        'Pn_St_Clab_V': [],    # lab_V
        'Pn_St_Capi_V': [],    # api_V
        'Pn_St_Cdors_V': [],   # dors_V

        # specification of type of syllable  (inclPhon)
        'Pn_St_C_V': [],
        'Pn_St_C_C_V': [],
       }
```

A4 The implementation of the phonological S-Pointer network

Concepts, lemmas, phonological syllables as well as motor plans and motor programs can be activated in all corresponding buffers in model 2, i.e., in all phonological, lemma, or concept buffers as well as in all motor plan and motor program buffers (see the buffers named in Fig. 5. and Fig. 6 as: P_..., L_..., C_..., …plan… and …prog…; see also Appendix D). If a concept, a lemma, a phonological syllable, a motor program or a motor plan is activated in one of these buffers this leads to a specific neural activation pattern, which exclusively represents that concept, lemma, etc. In mathematical terms, each neural activation pattern representing a specific neural activation state is specified as S-pointer. Sets of S-pointer are arbitrarily generated in a D-dimensional space (D = 256, see Kröger et al. 2020). The mathematics for defining and implementing sets of S-pointers is given in Eliasmith (2013) and Stewart and Eliasmith (2014). In model 2 we postulate relations between different sets of S-pointers, representing neural associations between concept and lemma buffers, between lemma and phonological syllable buffers, between phonological syllable and motor plan buffers as well as between motor plan and motor program buffers. This allows a direct coactivation of lemmas from concepts, of phonological forms from lemmas, of motor plans from phonological forms, and of motor programs from motor plans. This is implemented using hetero-associative memories which realize all details of learned neural associations between different buffers (ibid.). Because not all syllables are available as motor programs, some associations from the motor program buffer do not lead to any activation within the motor program buffer (case: syllables which still have to be learned).

In the case of the set of S-pointers generated for the phonological syllables, we in addition modeled internal relations between different syllable S-pointers by introducing the deep_phonos S-pointers within the structure feature subnetwork and by introducing relation type operators (see Appendix A). Thus, if two syllables “a” and “b” are defined as being related to each other (by introducing a specific relation type “typeC” (which is coded as an entry in the Python dictionary phonos as “phonos = { 'a': [(isRelatedWithByTypeC, 'b', …}”, see Appendix A2) this syllables “a” and “b” are similar with respect to the relation type “typeC”.

We have defined four relation types for our phonological network, i.e., “inclPhon”, “inclFeature”, “isSyllStruct”, and “isScoreType” (see Appendix A1). Analyzing the structure of the two python dictionaries phonos and phonos_deep (see Appendix A2 and A3) we find (i) that the relation type “inclPhon” separates CCV and CV syllables (i.e., builds up CCV syllables from CV syllables and thus separates types of syllables as defined in section 3.2 by the phonological structure feature “type of syllable”), (ii) that the relation type “isScoreType” separates all syllables with respect to different types of gesture scores (see definition of the phonological structure feature “type of gestural score” in section 3.2), (iii) that the relation type “isSyllStruct” separates different types of segments (see phonological structure feature “type of segments within a syllable” in section 3.2), and (iv) that the relation type “inclFeature” separates different types of features (see phonological structure feature “type of feature of a segment within a syllable).

Appendix B: model language word corpus for experiment 2

In the second experiment (section 3.2) a model language comprising 45 monosyllabic words (CV- and CCV-syllables) is used. The table below displays the arbitrary mapping of word meanings to the phonological representations of these monosyllabic words resulting from the phonological and semantic restrictions. The phonetic set of syllables is constructed symmetrically using a restricted set of speech sounds (consonants and vowels): CV-syllables: all eight consonants /b, d, g, p, t, k, m, n, l/ are combined with all three vowels /i, a, u/; CCV-syllables: six consonant clusters /bl, gl, pl, kl, gn, kn/ are combined with all three vowels. Word meanings are restricted to category nouns, verbs and adjectives which are typically learned in early phases of speech acquisition.

word meaning	phonological transcription
'Mom'	/ma/
'Dad'	/pa/
'Eat'	/ta/
'Drink'	/di/
'More'	/mu/
'Done'	/du/
'Stop'	/plu/
'Go'	/gu/
'Help'	/la/
'Open'	/pu/
'Walk'	/gla/
'Run'	/gna/
'Play'	/pla/
'Jump'	/kna/
'Baby'	/ba/
'Cow'	/ku/
'Fish'	/li/
'Duck'	/gli/
'Cat'	/ka/
'Dog'	/ga/
'Milk'	/gni/
'Cookie'	/kni/
'Water'	/kli/
'Juice'	/glu/
'Apple'	/blu/
'Banana'	/na/
'Book'	/klu/
'Ball'	/bla/
'Tree'	/ti/
'Sun'	/ni/
'Shoes'	/gnu/
'Hot'	/pi/
'Cold'	/kla/
'Down'	/bli/
'Please'	/pli/
'Thanks'	/tu/
'Me'	/mi/
'You'	/knu/
'Bye'	/bu/
'Hello'	/lu/
'Yes'	/ki/
'No'	/nu/
'Big'	/gi/
'Car'	/da/
'Bed'	/bi/

Appendix C: Implementation of the adaptation process for syllables

The adaptation of a motor program from an already trained similar syllable for generating the motor program of a new syllable is exemplified in our simulation model 2 for the case of new syllables, which exhibit the same type of gesture score than the similar syllable which is already activated in buffer P_plan_sim (see Fig. 5 and Fig. 6). The appearing activation in buffer M_score now activates the syllable oscillator of the similar syllable and thus specifies the points in time for starting and ending of each gesture as well as the target-reaching velocity of each gesture which is part of the new syllable. Thus, all time relevant parameters including the intergestural timing of all gestures building up the new syllable are adapted from the similar syllable (for details of the implementation of syllable oscillators in buffer M_score and gesture oscillators in buffer M_gest see Kröger et al. 2021). Because the type of gesture score is identical for the similar syllable and for the syllable under construction (for the new syllable) the remaining task is to specify each gesture target. This information can be assembled from the phonological information of the new syllable available in buffer M_plan (see Fig. 5 and Fig. 6). This gesture target information is forwarded to buffer M_score_targ and then adapted for gesture selection in buffer M_gest. The temporal parameters needed in buffer M_gest are adapted from the temporal information available in buffer M_score.

Appendix D: List of acronyms for buffers as used in simulation model 2:

Names of buffers and neuron ensembles used in simulation model 2 and appearing in Fig. 5 and Fig. 6 are listed in this table.

module	acronym of buffer	name of buffer
**control component**	in_con	input control buffer
	out_con	output control buffer
**cognitive processing**	C_cog_in	concept input buffer for cognitive processing
	C_cog_out	concept output buffer for cognitive processing
**perception pathway**	A_perc	auditory input buffer
	V_perc	visual input buffer
	P_perc	phonological form buffer (perception pathway)
	L_perc	lemma buffer (perception pathway)
	C_perc	concept buffer (perception pathway)
**production pathway**	C_prod	concept buffer (production pathway)
	L_prod	lemma buffer (production pathway)
	P_prod	phonological form buffer (production pathway)
	P_plan_sim	motor plan of similar syllable (using the S-pointer relations developed in the phonological S-pointer network)
	M_plan	motor plan
	M_prog	motor program
	M_socre	syllable oscillators of gesture score (see Kröger et al. 2011)
	M_score_targ	target information for all gestures
	M_gest	gesture oscillators (see Kröger et al. 2021)
